# Myocardium-derived angiopoietin-1 is essential for coronary vein formation in the developing heart

**DOI:** 10.1038/ncomms5552

**Published:** 2014-07-29

**Authors:** Yoh Arita, Yoshikazu Nakaoka, Taichi Matsunaga, Hiroyasu Kidoya, Kohei Yamamizu, Yuichiro Arima, Takahiro Kataoka-Hashimoto, Kuniyasu Ikeoka, Taku Yasui, Takeshi Masaki, Kaori Yamamoto, Kaori Higuchi, Jin-Sung Park, Manabu Shirai, Koichi Nishiyama, Hiroyuki Yamagishi, Kinya Otsu, Hiroki Kurihara, Takashi Minami, Keiko Yamauchi-Takihara, Gou Y. Koh, Naoki Mochizuki, Nobuyuki Takakura, Yasushi Sakata, Jun K. Yamashita, Issei Komuro

**Affiliations:** 1 Department of Cardiovascular Medicine, Osaka University Graduate School of Medicine, 2-2, Yamadaoka, Suita, Osaka 565-0871, Japan; 2 Precursory Research for Embryonic Science and Technology (PRESTO), Japan Science Technology Agency, 4-1-8, Honcho, Kawaguchi, Saitama 332-0012, Japan; 3 Laboratory of Stem Cell Differentiation, Institute for Frontier Medical Sciences/Department of Cell Growth & Differentiation, Center for iPS Cell Research and Application (CiRA), Kyoto University, 53, Shogoin-Kawahara-cho, Sakyo-ku, Kyoto 606-8507, Japan; 4 Department of Signal Transduction, Research Institute for Microbial Diseases, Osaka University, 3-1, Yamadaoka, Suita, Osaka 565-0871, Japan; 5 Department of Physiological Chemistry and Metabolism, Graduate School of Medicine, University of Tokyo, 7-3-1, Hongo, Bunkyo-ku, Tokyo 113-0033, Japan; 6 National Research Laboratory of Vascular Biology and Stem Cells, Graduate School of Medical Science and Engineering, Korea Advanced Institute of Science and Technology (KAIST), 373-1, Guseong-dong, Daejeon 305-701, Korea; 7 Department of Bioscience, National Cerebral and Cardiovascular Center Research Institute, 5-7-1, Fujishirodai, Suita, Osaka 565-8565, Japan; 8 Department of Pediatrics, Keio University School of Medicine, 35, Shinano-machi, Shinjuku-ku, Tokyo 160-8582, Japan; 9 Cardiovascular Division, King’s College London, 25, Coldharbour Lane, London SE5 9NU, UK; 10 Laboratory for Vascular Biology, Research Center for Advanced Science and Technology, University of Tokyo, 4-6-4, Komaba, Meguro-ku 153-8904, Tokyo, Japan; 11 Department of Cell Biology, JST-CREST, National Cerebral and Cardiovascular Center Research Institute, 5-7-1, Fujishirodai, Suita, Osaka 565-8565, Japan; 12 Department of Stem Cell Differentiation, Institute for Frontier Medical Sciences, Kyoto University, 53, Shogoin-Kawahara-cho, Sakyo-ku, Kyoto 606-8507, Japan; 13 Department of Cardiovascular Medicine, Graduate School of Medicine, University of Tokyo, 7-3-1, Hongo, Bunkyo-ku, Tokyo 113-0033, Japan; 14 Core Research for Evolutional Science and Technology (CREST), Japan Science Technology Agency, 4-1-8, Honcho, Kawaguchi, Saitama 332-0012, Japan

## Abstract

The origin and developmental mechanisms underlying coronary vessels are not fully elucidated. Here we show that myocardium-derived angiopoietin-1 (Ang1) is essential for coronary vein formation in the developing heart. Cardiomyocyte-specific *Ang1* deletion results in defective formation of the subepicardial coronary veins, but had no significant effect on the formation of intramyocardial coronary arteries. The endothelial cells (ECs) of the sinus venosus (SV) are heterogeneous population, composed of APJ-positive and APJ-negative ECs. Among these, the APJ-negative ECs migrate from the SV into the atrial and ventricular myocardium in Ang1-dependent manner. In addition, Ang1 may positively regulate venous differentiation of the subepicardial APJ-negative ECs in the heart. Consistently, *in vitro* experiments show that Ang1 indeed promotes venous differentiation of the immature ECs. Collectively, our results indicate that myocardial Ang1 positively regulates coronary vein formation presumably by promoting the proliferation, migration and differentiation of immature ECs derived from the SV.

## Abstract (web summary)

The secreted ligand Angiopoietin-1 is essential for embryonic blood vessel development and adult vascular homeostasis. Here the authors show, using conditional knockout mice, that myocardium-derived Angiopoietin-1 is required for the formation of coronary veins, but not arteries.

## Graphical abstract

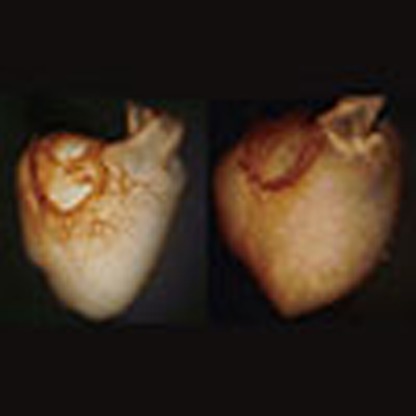

As the heart develops and the chamber walls thicken during embryonic development, passive diffusion of oxygen and nutrients is replaced by a vascular plexus, which is remodelled and expands to form a mature coronary vascular system^1^,^2^. The coronary arteries and veins ensure the continued development of the heart and progressively increase cardiac output towards birth. Elucidating the cellular and molecular signals involved in vascularizing the embryonic heart would provide significant insights into adult heart disease and tissue regeneration. However, many aspects of the developmental origins of coronary endothelial cells (ECs) and the specific signals determining their fate have not been fully elucidated to date^2^,^3^.

The heart is arranged in three layers: the endocardium, myocardium and epicardium. The epicardium is the outermost layer and is derived from the proepicardium located outside, but close to the heart. The myocardium is the central layer, within which the coronary vasculature develops. It is unclear whether proepicardium/epicardial cells contribute significantly to coronary EC formation in mammals, although some coronary ECs in avian species are derived from proepicardial cells^4^,^5^,^6^. However, more recent studies in mammals demonstrated that epicardial cells generate coronary vascular smooth muscle cells but not coronary ECs^7^,^8^. A recent report showed that the coronary vessels in mammals are primarily derived from a common origin, the differentiated venous ECs in the sinus venosus (SV), a major vein located just above the developing liver that returns blood to the embryonic heart^9^. According to that report, the sprouting venous ECs dedifferentiate when they migrate over or invade the myocardium. The intramyocardial invading ECs redifferentiate into arteries, whereas the ECs proceeding along the subepicardial layer of the heart redifferentiate into veins^9^. Another recent study reported that the Semaphorin3D/Scleraxis lineage-traced proepicardial cells, which traverse through SV endothelium en route to the heart and/or transiently contribute to the endocardium, differentiate into the coronary ECs^10^. A more recent report suggested that endocardial ECs generate the endothelium of coronary arteries through myocardial–endothelial signalling by vascular endothelial growth factor-A (VEGF-A) and vascular endothelial growth factor receptor 2 (VEGFR2)^11^. These findings suggest that coronary arteries and veins have distinct origins and are formed by different molecular mechanisms. Especially, the molecular mechanisms of coronary vein formation have been elusive to date.

Angiopoietin-1 (Ang1) is a member of the angiopoietin family of growth factors and is a major ligand for Tie2, a tyrosine kinase receptor primarily expressed on ECs^12^,^13^. Ang1/Tie2 signalling is required for EC quiescence, pericyte recruitment and the formation of stable vessels^12^. The Ang1/Tie2 signalling pathway is critical for normal development, since conventional *Ang1* or *Tie2* knockout mice exhibit embryonic lethality between E9.5 and E12.5, with similar abnormal vascular phenotypes and loss of heart trabeculation^14^,^15^. We previously found that neuregulin-1(NRG-1)/ErbB signalling is essential for cardiac homeostasis presumably via Ang1 secreted from cardiomyocytes^16^. Thus, we investigated the role of myocardial-derived Ang1 in coronary vessel formation and cardiac homeostasis by creating cardiomyocyte-specific Ang1-knockout mice.

In the present study, we show that myocardium-derived Ang1 is indispensable for coronary vein formation in the developing heart. Cardiomyocyte-specific *Ang1* deletion results in defective formation of the subepicardial coronary veins, but does not affect the formation of the intramyocardial coronary arteries. The ECs of the SV consist of two heterogeneous populations, namely APJ-positive and APJ-negative ECs. Among these, the APJ-negative ECs migrate from the SV into the atrial and ventricular myocardium in Ang1-dependent fashion. In addition, Ang1 promotes venous differentiation of the subepicardial APJ-negative ECs in the heart. Furthermore, *in vitro* experiments using the Flk1-positive immature endothelial progenitor cells demonstrate that Ang1 indeed promotes venous differentiation. Taken together, these findings suggest that myocardial Ang1 has an essential role in coronary vein formation presumably by promoting the proliferation, migration and differentiation of immature ECs derived from the SV.

## Results

### Myocardial deletion of *Ang1* results in embryonic lethality

We hypothesized that myocardium-derived Ang1 is essential for heart development by mediating coronary vessel formation. To test this hypothesis, we generated cardiomyocyte-specific *Ang1-*knockout (Ang1CKO) mice using the *Cre-loxP* system. We created an *Ang1*^*flox*^ allele by introducing two *loxP* sites into introns flanking exon 1, which encodes part of the signal sequence^17^. To generate Ang1CKO mice, we crossed *Ang1*^*flox/flox*^ mice with *α-MHC-Cre* transgenic mice^16^. We confirmed the expected genetic recombination at the *Ang1* locus in the heart, but not in the head of Ang1CKO (*Ang1*^*flox/flox*^*; α-MHC-Cre*) embryos (Supplementary Fig. 1a). We also confirmed that *Ang1* mRNA was ablated from the ventricles of Ang1CKO embryos compared with that of control (*Ang1*^*flox/flox*^) embryos by whole-mount *in situ* hybridization (Supplementary Fig. 1b)^18^. In addition, *Ang1* mRNA expression was significantly reduced in the hearts of Ang1CKO embryos compared with those of control embryos at E8.5-E10.5 as assessed by quantitative reverse transcription –PCR (qRT–PCR; Supplementary Fig. 1c). Consistently, we confirmed that the Cre-mediated recombination at E8.5 and E9.5 through crossing *α-MHC-Cre* mice with enhanced GFP reporter mice (*CAG-CAT-EGFP* mice) (Supplementary Fig. 1d). No live Ang1CKO mice were obtained, and Ang1CKO embryos died in uterus between E12.5 and E14.5, slightly later than conventional Ang1-knockout (Ang1KO) mice, which die at E12.5 (Supplementary Table 1)^15^,^19^.

### Ang1CKO embryos show defects in coronary vein formation

**Figure 1:**
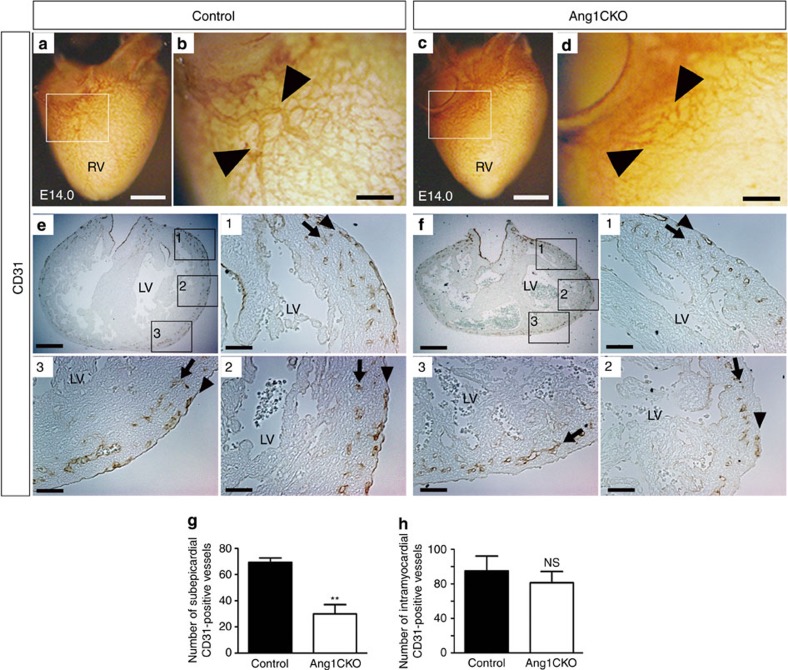
Myocardial Ang1 is crucial for subepicardial coronary vessel formation. (**a**–**d**) Whole-mount immunostaining of embryonic hearts at E14.0 with anti-CD31 antibody. Myocardial Ang1 was required for subepicardial CD31-positive vessel remodelling (**a**,**b**: arrowheads in magnified image of inset). The CD31-positive vessel formation was impaired in the ventricles of Ang1CKO embryos (**c**,**d**: arrowheads in magnified image of inset). (**e**,**f**) Sectioned analysis of the whole-mount immunostained embryonic heart. Subepicardial CD31-positive vessels were detected uniformly in all the sections from control embryo ventricles (**e**), whereas the density of subepicardial CD31-positive vessels decreased gradually from the dorsal (area 1) to the ventral side (area 3) in Ang1CKO embryos (**f**). Arrows and arrowheads indicate the intramyocardial CD31-positive vessels and subepicardial CD31-positive vessels, respectively. Area 1, dorsal side; area 2, lateral side; area 3, ventral side of the ventricles. (**g**,**h**) Quantification of the number of subepicardial and intramyocardial CD31-positive vessels in the transverse section including inflow-tract of ventricle from E14.0 (*n*=3). Scale bars, 400 μm in **a**,**c**; 100 μm in **b**,**d**; 200 μm in **e**,**f**; and 50 μm in magnified images of insets 1–3. LV, left ventricle; RV, right ventricle. Values are shown as means±s.e.m. for three separate experiments. Student’s *t*-test was used to analyse differences. **P<0.01 compared with control. NS, not significant.

To examine the effect of myocardial deletion of Ang1 on the coronary vessel formation, we performed a histological analysis of the hearts of Ang1CKO embryos. Whole-mount CD31-immunostaining revealed that Ang1CKO embryos exhibited impaired subepicardial coronary vessel remodelling compared with control embryos (Fig. 1a–d, Supplementary Fig. 2a–d). The whole-mount stained samples were then sectioned for analysis. The subepicardial CD31-positive vessel formation was specifically disturbed in Ang1CKO embryos (Fig. 1e,f), whereas the intramyocardial CD31-positive vessel formation was almost similar in control and Ang1CKO embryos (Fig. 1e,f,h). Although the subepicardial CD31-positive vessels were detected uniformly from the dorsal to ventral side in the ventricles of control embryos (Fig. 1e), the number of subepicardial CD31-positive vessels gradually decreased from the dorsal (Fig. 1f, area 1) to ventral side (Fig. 1f, area 3) in the ventricles of Ang1CKO embryos. The number of subepicardial CD31-positive coronary vessels in the transverse section containing the inflow-tract area in Ang1CKO embryos was indeed smaller by 57% than that of control (Fig. 1g), indicating that the cardiomyocyte-specific deletion of *Ang1* resulted in the impaired formation of subepicardial CD31-positive coronary vessels in the heart.

**Figure 2:**
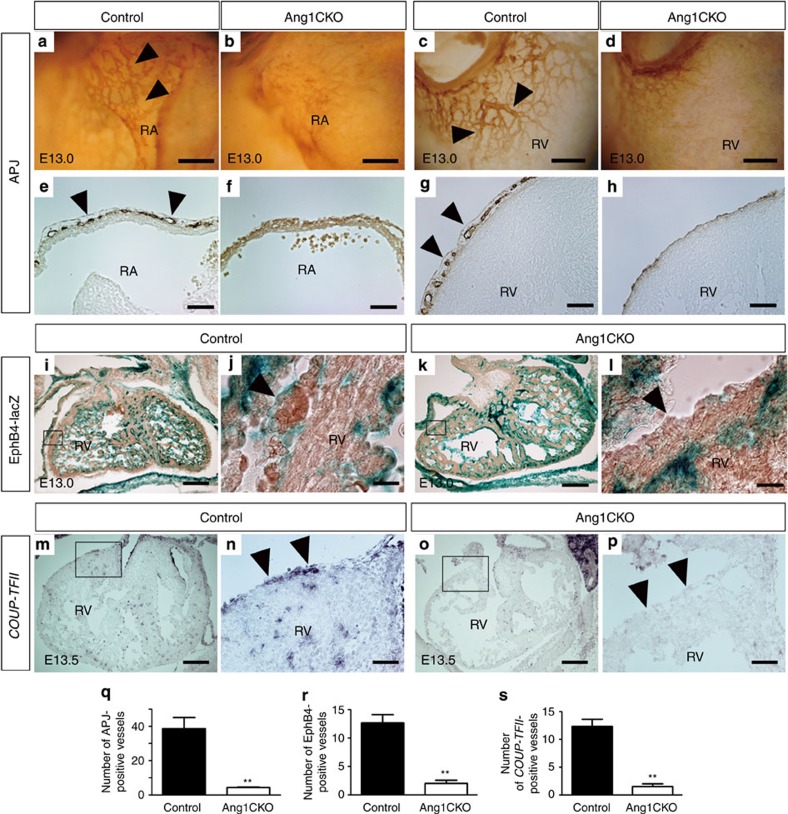
Myocardial Ang1 is essential for coronary vein formation. (**a**–**d**) Whole-mount immunostaining of embryonic hearts with anti-APJ antibody. APJ-positive coronary veins were observed on the surfaces of both the RA (**a**, arrowheads) and RV (**c**, arrowheads) of control embryos, but not on the RA (**b**) or RV (**d**) of Ang1CKO embryos. (**e**–**h**) Sectioned analyses of the whole-mount immunostained embryonic hearts revealed subepicardial APJ-positive coronary veins with vessel-like structures in the RA (**e**, arrowheads) and RV (**g**, arrowheads) of control, but not Ang1CKO embryos (**f**,**h**). (**i**–**l**; Note: for the experiment presented in **i**–**l**, both ‘control’ and ‘Ang1CKO’ mice contained the *EphB4 tau-lacZ* knockin allele; see Results.) EphB4-lacZ-positive signals in the hearts of control and Ang1CKO embryos at E13.0. EphB4-lacZ-positive subepicardial coronary veins were observed in control (**i**,**j**, arrowhead), but not Ang1CKO embryos (**k**,**l**, arrowhead). EphB4-lacZ-positive signals were also detected in the endocardial endothelium in both control and Ang1CKO embryos. (**m**–**p**) Expression patterns of *COUP-TFII* in the hearts at E13.5. *COUP-TFII*-positive subepicardial coronary veins were observed in control (**m**,**n**, arrowhead), but not in Ang1CKO embryos (**o**,**p**, arrowhead). (**q**–**s**) Quantification of the number of subepicardial APJ (**q**, E13.5), EphB4-lacZ (**r**, E13.0) and *COUP-TFII* (**s**, E13.5) -positive vessels in the transverse section including inflow-tract of ventricle (*n*=3). (**r**) The number of the EphB4-positive vessels especially with vessel-like structures or with red blood cells were quantified. Scale bars, 100 μm in **a**–**h**; 300 μm in **i**,**k**,**m**,**o**; 25 μm in **j**,**l**; and 75 μm in **n**,**p**. RA, right atrium; RV, right ventricle. Values are shown as means±s.e.m. for three separate experiments. Student’s *t*-test was used to analyse differences. **P<0.01 compared with control.

The subepicardial and intramyocardial CD31-positive vessels are reported to give rise to the coronary veins and arteries, respectively^9^,^20^. Thus, our findings suggest that the cardiomyocyte-specific deletion of *Ang1* may lead to defective formation of the subepicardial coronary veins. To characterize the lost vessels in the hearts of Ang1CKO embryos, we examined the expression of APJ, which is confined to the veins, by whole-mount immunostaining^21^,^22^. Intriguingly, APJ-positive subepicardial venous vessels were observed in both atria and ventricles of control embryos, but not in those of Ang1CKO embryos (Fig. 2a–d, Supplementary Fig. 3a,b). Analysis of the sectioned samples clearly revealed subepicardial APJ-positive vessel structures in the control but not in the Ang1CKO embryos (Fig. 2e–h, Supplementary Fig. 3c,d), suggesting that cardiomyocyte-derived Ang1 is required for the formation of subepicardial APJ-positive mature coronary veins.

To examine the expression of Eph receptor B4 (EphB4), another venous endothelial marker, in the Ang1CKO embryos, we created *Ang1flox/flox; α-MHC-Cre(+)*; *EphB4 tau-lacZ(+)* by crossing *Ang1+/flox; α-MHC-Cre-*TG mice with *EphB4 tau-lacZ* knockin mice, which express the *lacZ* reporter gene in the venous ECs^23^. EphB4-lacZ-positive signals were observed in the subepicardial region of the hearts of control embryos at E13.0, but not in those of Ang1CKO embryos (Fig. 2i–l, Supplementary Fig. 3e–h).

Chicken ovalbumin upstream promoter-transcription factor II (COUP-TFII), is reported to be a critical transcription factor for the venous identity of ECs^24^,^25^. *In situ* hybridization revealed that the expression of *COUP-TFII* is observed in the subepicardial region of the hearts of control embryos at E13.5, but not in those of Ang1CKO embryos (Fig. 2m–p). The numbers of subepicardial differentiated coronary veins identified by APJ-positive vessels, EphB4-lacZ-positive vessels and *COUP-TFII*-positive vessels in Ang1CKO embryos were reduced by 84–89% compared with those of control embryos (Fig. 2q–s). In addition, the quantification revealed that the number of subepicardial mature coronary veins positive for the above venous markers in the ventricles was much less than that of subepicardial immature CD31-positive vessels. Taken together, these findings suggest that cardiomyocyte-specific deletion of *Ang1* impairs the formation of subepicardial coronary veins.

### Ang1CKO embryos show no defects in coronary artery formation

**Figure 3:**
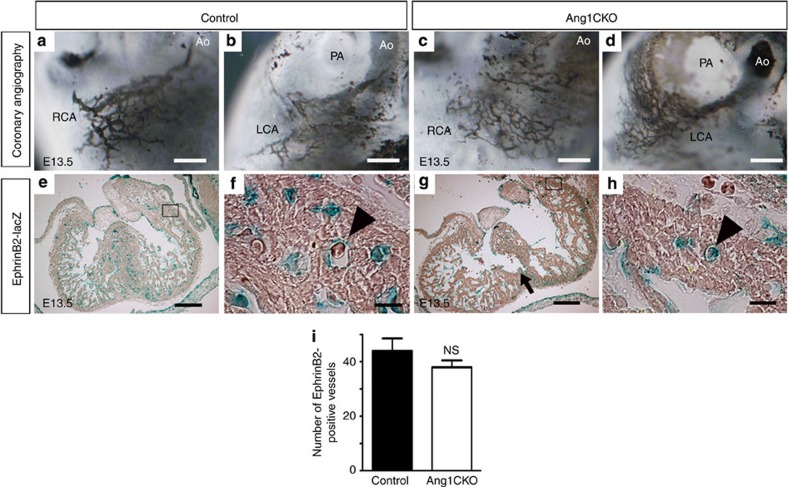
Myocardial Ang1 is dispensable for coronary artery formation. (**a**–**d**) Coronary angiography by ink injection into the hearts of control (**a**,**b**) and Ang1CKO embryos (**c**,**d**) at E13.5. (**e**–**h**; Note: for the experiment presented in **e**–**h**, both ‘control’ and ‘Ang1CKO’ mice contained the *EphrinB2 tau-lacZ* knockin allele; see Results.) EphrinB2-lacZ-positive signals in the hearts of control and Ang1CKO embryos at E13.5. EphrinB2-lacZ-positive intramyocardial coronary arteries (containing red blood cells) were similarly observed in control (**e**,**f**, arrowhead) and Ang1CKO embryos (**g**,**h**, arrowhead). The arrow indicates impaired formation of the interventricular septum in Ang1CKO embryos. (**i**) Quantification of the number of RV+LV free wall EphrinB2-lacZ-positive vessels in the transverse section including inflow-tract of ventricle from E13.5 (*n*=3). Scale bars, 100 μm in **a**–**d**; 300 μm in **e**,**g**; and 50 μm in **f**,**h**. Ao, aorta; LCA, left coronary artery; RCA, right coronary artery. Values are shown as means±s.e.m. for three separate experiments. Student’s *t*-test was used to analyse differences. NS, not significant.

Next, we examined the effect of cardiomyocyte-specific *Ang1* deletion on the formation of coronary arteries. Since the coronary arteries penetrate the aorta at E13.5 (ref. 1)^1^, we performed coronary angiography in E13.5 embryos using an ink injection technique (see Methods)^26^. The results revealed that there was no significant difference in coronary artery formation between the control and Ang1CKO embryos (Fig. 3a–d).

To confirm the expression of arterial endothelial marker EphrinB2 in the Ang1CKO embryos, we created *Ang1flox/flox; α-MHC-Cre(+)*; *EphrinB2 tau-lacZ(+)* mice by crossing *Ang1+/flox; α-MHC-Cre-*TG mice with *EphrinB2 tau-lacZ* knockin mice, which express the *lacZ* reporter gene in arterial ECs^27^. EphrinB2-lacZ-positive signals were comparably observed in the intramyocardial layers of control and Ang1CKO embryos at E13.0 (Fig. 3e–h). The number of EphrinB2-lacZ-positive vessels in Ang1CKO embryos was almost similar to that in control embryos (Fig. 3i). These findings suggest that cardiomyocyte-specific deletion of *Ang1* did not affect the formation of the intramyocardial coronary arteries.

### Myocardial deletion of *Ang1* reduces venous marker expression

**Figure 4:**
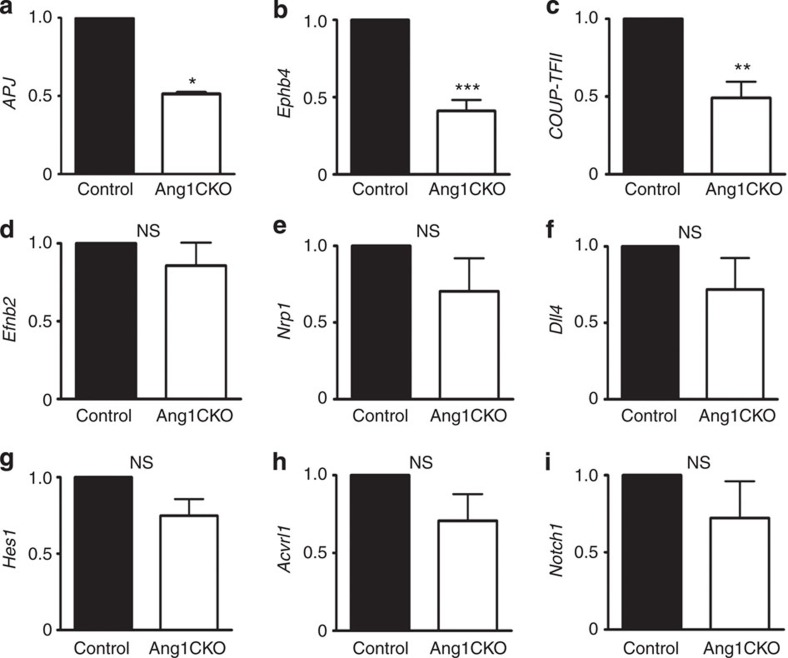
The expression levels of venous marker genes are significantly lower in the hearts of Ang1CKO embryos than in those of control. (**a**–**i**) Quantitative expression analysis of venous and arterial marker mRNAs in the ventricles at E12.5-E13.0 (normalized to *GAPDH* mRNA; *n*=3). The expression levels of venous marker genes such as *APJ* (**a**), *Ephb4* (**b**)*, COUP-TFII* (**c**), were significantly reduced in Ang1CKO embryos compared with control. However, the expression levels of arterial marker genes such as *EfnbB2* (**d**), *Nrp1* (**e**), *Dll4* (f), *Hes1* (**g**), *Acvrl1* (**h**) and *Notch1* (**i**) were not significantly affected in Ang1CKO embryos compared with control. Values are shown as means±s.e.m. for three separate experiments. Student’s *t*-test was used to analyse differences. *P<0.05, **P<0.01, ***P<0.001 compared with control. NS, not significant.

To confirm the defect in coronary vein formation in Ang1CKO embryos quantitatively, we examined the expression levels of venous marker genes including *APJ, Ephb4* and *COUP-TFII*^28^. The expression levels of these mRNAs were significantly reduced in the hearts of Ang1CKO embryos compared with those of controls (Fig. 4a–c). In contrast, the expression levels of arterial marker genes such as *Efnb2, neuropilin-1 (Nrp1), Delta-like 4 (Dll4), hairy and enhancer of split 1 (Hes1), activin receptor-like kinase 1 (Acvrl1)* and *Notch1* were not significantly affected in the hearts of Ang1CKO embryos compared with those of controls (Fig. 4d–i). These data indicate that myocardial Ang1 is specifically involved in the formation of coronary veins, but not coronary arteries.

### Ang1CKO embryos display impaired development of myocardium

Through the analysis of Ang1CKO embryos, we noticed that Ang1CKO embryos display impaired development of the hearts. The thicknesses of compact layers in Ang1CKO embryos were significantly thinner by approximately 40% than those in control embryos (Supplementary Fig. 4a–d). In addition, Ang1CKO embryos displayed a mild defect in trabeculation compared with control embryos (Supplementary Fig. 4a,b). Therefore, we next performed immunostaining with anti-phospho-histone H3 (pHH3) antibody. Ang1CKO embryos display impaired proliferation of cardiomyocytes compared with control embryos (Supplementary Fig. 4e–g). We previously reported that NRG-1/ErbB signalling is essential for cardiac homeostasis presumably in part via secretion of Ang1 from cardiomyocytes^16^. NRG-1 is an EGF-family growth factor, which is essential for myocardial growth and trabeculation through the activation of ErbB receptors during embryogenesis^29^. Thus, we examined whether myocardial deletion of Ang1 might affect the expression level of NRG-1 in the hearts. We found that the mRNA level of *Nrg-1* in the hearts of Ang1CKO embryos was significantly lower than in those of control embryos (Supplementary Fig. 4h). Therefore, the reduced myocardial growth of Ang1CKO embryos might be partly attributed to the reduced expression of *Nrg-1* gene in the hearts of Ang1CKO embryos. In addition, we also found that the formation of interventricular septum was significantly impaired in all Ang1CKO embryos compared with control at E13.5-E14.0 presumably due to the impaired myocardial growth (Fig. 3e,g, Supplementary Fig. 4a,b). Taken together, these findings indicate that the embryonic lethality of Agn1CKO embryos between E12.5 and E14.5 might be ascribed to the combinatorial defects in both coronary vein formation and myocardial growth.

### Ang1 is predominantly expressed in the atria and ventricles

We next examined the expression pattern of Ang1 in the embryonic heart during coronary vessel formation. We generated an *Ang1-mCherry* reporter mouse as described in Methods, since commercial antibodies were not available to detect the endogenous expression of Ang1 in murine tissue. Immunostaining with an anti-DsRed antibody in the cryosectioned samples of Ang1-mCherry reporter mice demonstrated that Ang1 was strongly expressed in the atria and ventricles to a similar extent at E11.5 (Supplementary Fig. 5a). At E13.5, Ang1 expression was confined to the atria and ventricular trabeculae (Supplementary Fig. 5b).

**Figure 5:**
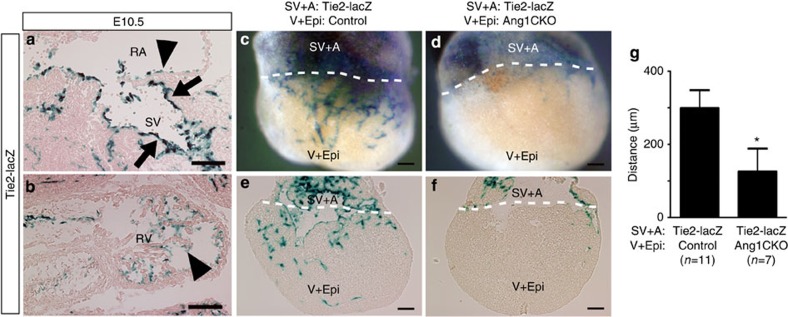
Ang1 derived from the ventricular myocardium attracts Tie2-positive ECs from the SV. (**a**,**b**) X-gal staining of *Tie2-lacZ* transgenic mice at E10.5. Tie2-positive signals were observed most strongly in the SV (arrows), and to a much lesser extent in the endocardium of both the RA and RV (arrowheads). (**c**–**f**) Analysis of coronary vessel sprouting *in vitro.* The SV and atrium (SV+A) resected from *Tie2-LacZ* transgenic embryos were recombined with the ventricle and epicardium (V+Epi) resected from either control or Ang1CKO embryos at E10.5, cultured for 72 h at 37 °C, and subjected to whole-mount staining with X-gal (**c**,**d**). Tie2-lacZ-positive coronary sprouts formed when recombined with the V+Epi from control (**c**), but not with the V+Epi from Ang1CKO embryos (**d**). Whole-mount X-gal-stained samples were sectioned (**e**,**f**). The migratory distances from the combined atrioventricular borderline (dotted line) to the forefront of the Tie2-lacZ-positive signals in the ventricles were measured. The mean migratory distance of each group is shown in **g**. Scale bars, 100 μm. A, atrium; Epi, epicardium; RV, right ventricle; SV, sinus venosus; V, ventricle. Values are shown as means±s.e.m.. Student’s *t*-test was used to analyse differences. *P<0.05 compared with control.

We next evaluated the expression of Tie2 by X-gal staining using *Tie2-lacZ* transgenic mice^30^. At E10.5, the strongest Tie2-lacZ signal in the cardiovascular system of these mice was detected in the SV (Fig. 5a,b). Tie2-lacZ signals were also observed in the endocardial endothelium of the atria and ventricles at E10.5, but were markedly weaker than those in the SV (Fig. 5a,b), indicating that Ang1 may be involved in the migration of Tie2-positive ECs of the SV toward the myocardium.

### Myocardial Ang1 attracts Tie2-positive ECs from SV

Previous study suggested that the ECs of the SV are one of the most plausible candidate sources of coronary ECs^9^. In addition, Tie2 was most strongly expressed in the ECs of the SV at E10.5, when the ECs of the SV begin to invade the atrium (Fig. 5a,b). Thus, we hypothesized that myocardial Ang1 might attract the ECs of the SV. Therefore, we examined whether myocardial Ang1 is indeed responsible for the migration of ECs from the SV into the ventricular myocardium by using a cardiac organ culture system modified to enable the detection of Tie2-lacZ-positive ECs migrating from the SV^9^,^30^. Developing hearts were isolated from *Tie2-lacZ* transgenic mice, *Ang1flox/flox* (control) mice or Ang1CKO mice at E10.5, after the proepicardium has spread over the heart surface to form the epicardium but before any coronary sprouts are present. Since Tie2-lacZ was expressed in the endocardial endothelium of the embryonic heart, Tie2-lacZ signals were detected throughout the embryonic heart when the intact hearts were resected from *Tie2-lacZ* transgenic mice, cultured for 72 h and stained with X-gal (Supplementary Fig. 6a). On the contrary, no Tie2-lacZ-positive signals were detected throughout the embryonic heart resected from wild-type (control) embryos (Supplementary Fig. 6b). Therefore, the hearts were dissected to separate the ventricles with their epicardial covering (V+Epi) from the SV and atria (SV+A). When the SV+A or V+Epi resected from Tie2-lacZ mice were cultured separately for 72 h and stained with X-gal, Tie2-lacZ-positive signals were observed in the atria or ventricle in a similar patchy fashion (Supplementary Fig. 6c,d).

The SV+A resected from *Tie2-lacZ* transgenic mice were combined with the V+Epi resected from either control or Ang1CKO mice, and then cultured for 72 h and stained with X-gal to determine whether Tie2-lacZ-positive coronary vessels had sprouted and migrated into the ventricles (Fig. 5). When the SV+A resected from *Tie2-lacZ* mice was cultured with the V+Epi of control mice, Tie2-lacZ-positive coronary vessels were detected on the V+Epi (Fig. 5c,e; 7 of 11, 64%). However, when the SV+A resected from *Tie2-lacZ* mice were cultured with the V+Epi of Ang1CKO mice, the formation of Tie2-lacZ-positive coronary vessels on the V+Epi was disturbed (Fig. 5d,f; 2 of 7, 29%). Consistent with these findings, the mean migratory distance from the border between the atrium and ventricle to the leading edge of the Tie2-positive ECs in the ventricle was significantly longer in the culture of the *Tie2-lacZ* atrium with the control ventricle than in that of the *Tie2-lacZ* atrium with the Ang1CKO ventricle (Fig. 5g). Collectively, these experiments suggest that Ang1 derived from the ventricular myocardium might be involved in attraction of the Tie2-positive ECs from the SV towards the ventricular myocardium.

### The ECs of SV consist of APJ-positive and APJ-negative cells

**Figure 6:**
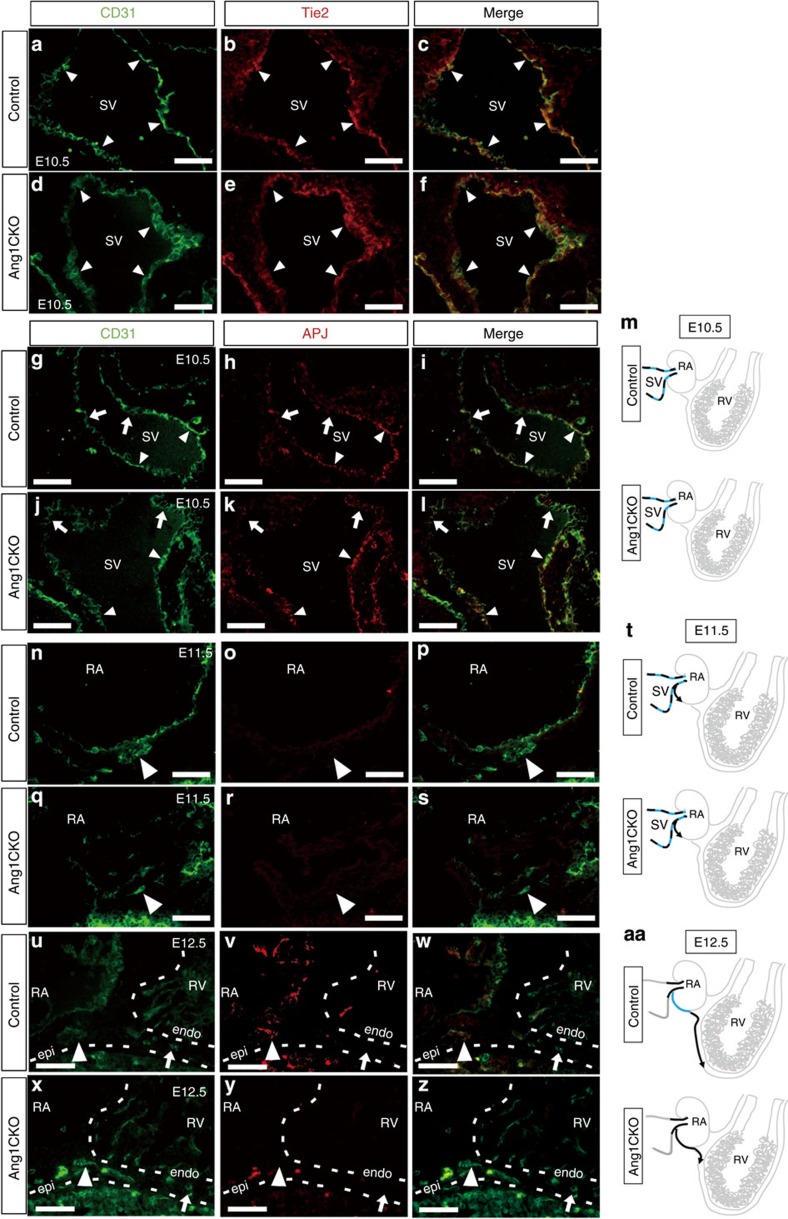
APJ-negative ECs sprout off from the SV and migrate into the embryonic atria and ventricles. (**a**–**f**) Sagittal section through the SV of control (**a**–**c**) and Ang1CKO embryos (**d**–**f**) at E10.5 immunostained for CD31 (green) and Tie2 (red). CD31 and Tie2 were uniformly expressed in the ECs of the SV (arrowheads). (**g**–**l**) Sagittal section through the SV of control (**g**–**i**) and Ang1CKO embryos (**j**–**l**) at E10.5 immunostained for CD31 (green) and APJ (red). APJ-negative ECs were detected among the CD31-positive ECs in both control and Ang1CKO embryos (arrows). ECs in the SV expressing both CD31 and APJ were similarly observed in control and Ang1CKO embryos (arrowheads). (**m**) Schematic illustrations of the SV at E10.5 showing that the ECs were heterogeneous for APJ expression. (**n**–**s**) Sagittal sections through the right atrium (RA) of control (**n**–**p**) and Ang1CKO embryos (**q**–**s**) at E11.5 immunostained for CD31 (green) and APJ (red). (**t**) Schematic illustrations of the RA at E11.5 showing that all of the invading ECs were negative for APJ in both control and Ang1CKO embryos. (**u**–**z**) Sagittal section through the RA and right ventricle (RV) of control (**u**–**w**) and Ang1CKO embryos (**x**–**z**) at E12.5 immunostained for CD31 (green) and APJ (red). The CD31-positive ECs invading the RV did not express APJ in either control or Ang1CKO embryos (arrow). In contrast, APJ was expressed in all of the CD31-positive ECs in the RA of control, but not Ang1CKO embryos (arrowheads). (**aa**) Schematic illustration of the RA and RV at E12.5 summarizing the expression of APJ. Blue line, APJ-positive ECs; black line, APJ-negative ECs. Scale bars, 50 μm. RA, right atrium; RV, right ventricle; SV, sinus venosus.

The SV is considered one of the most plausible sites of origin for coronary vein ECs^9^,^11^. Therefore, we examined the expression patterns of several EC markers in the SV. We performed double immunostaining with anti-CD31 and anti-Tie2 antibodies. Consistent with the results obtained from the X-gal staining of *Tie2-lacZ* transgenic mice, all of the CD31-positive ECs in the SV were positive for Tie2 in control embryos (Fig. 6a–c). Furthermore, the expression pattern of Tie2 in Ang1CKO and control embryos was quite similar (Fig. 6d–f), suggesting that Tie2 is uniformly expressed in the ECs of the SV in both control and Ang1CKO embryos. In addition, VEGFR2 immunostaining colocalized with that of Tie2 in the SV of control and Ang1CKO embryos (Supplementary Fig. 7a–f), indicating that VEGFR2 is also uniformly expressed in the ECs of the SV. We also performed X-gal staining of *EphrinB2-lacZ* knockin mice at E10.5 and confirmed that the arterial endothelial marker EphrinB2 is not expressed in the SV at E10.5 (Supplementary Fig. 7g–i). These data suggest that CD31, Tie2 and VEGFR2 are all expressed uniformly in the ECs of the SV.

In clear contrast, APJ was not uniformly expressed in the ECs of the SV in either control or Ang1CKO embryos (Fig. 6g–l). This finding indicates that the ECs in the SV at E10.5 consist of two populations, namely the APJ-positive and APJ-negative ECs (Fig. 6m).

### The APJ-negative ECs migrate from SV into myocardium

We next addressed the characteristics of the CD31-positive ECs migrating from the SV into the atrium. Intriguingly, APJ-negative ECs were exclusively detected in the coronary sprouts in the atria of both control and Ang1CKO embryos at E11.5 (Fig. 6n–t). We also confirmed that APJ-positive vessels were not detected in the atrium of either control or Ang1CKO embryos at E11.5 by whole-mount immunostaining with an anti-APJ antibody (Supplementary Fig. 7j–m). However, at E12.5, APJ was expressed in the atrial CD31-positive vessels in control embryos, but not in Ang1CKO embryos (Fig. 6u–z, arrowheads). These data are consistent with the data obtained from whole-mount immunostaining with the anti-APJ antibody at E13.0 (Fig. 2a,b). Taken together, these findings suggest that Ang1 secreted from the atrial myocardium promotes the upregulation of APJ and the venous differentiation of ECs sprouting from the SV into the atrium of control embryos (Fig. 6aa).

The expression of APJ in the vessels migrating from the atrium into the ventricles was not detected in control or Ang1CKO embryos at E12.5 (Fig. 6u–z, arrow). These data indicate that the APJ-negative ECs migrate from the SV into the atrium at E11.5 (Fig. 6t) and subsequently into the ventricle at E12.5, followed by the appearance of APJ-positive, mature ECs in the atrium (Fig. 6aa). Whole-mount immunostaining analysis of the wild-type embryos revealed the emergence of APJ-positive vessels on the ventricular surface on and after E13.5 (Supplementary Fig. 2e–h), one day later than the emergence of CD31-positive vessels (Supplementary Fig. 2a–d). These findings indicate that the APJ-negative ECs precede the appearance of the APJ-positive mature venous ECs in the ventricle.

**Figure 7:**
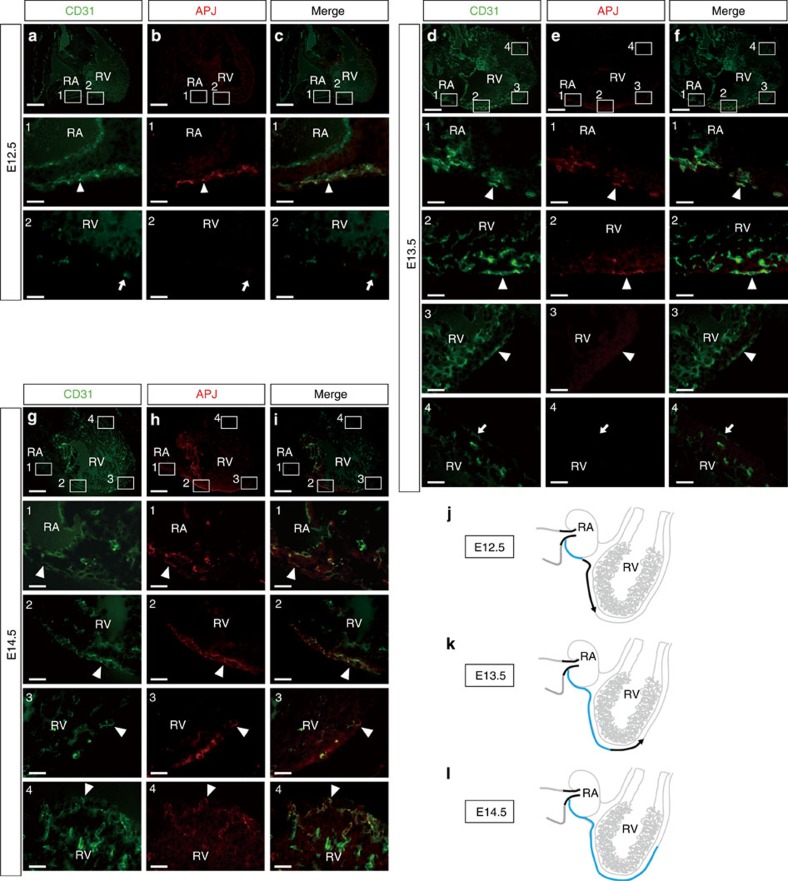
APJ-negative ECs precede APJ-positive ECs on the ventricle surface. Sagittal sections through the RA and RV of wild-type embryos at E12.5 (**a**–**c**), E13.5 (**d**–**f**), E14.5 (**g**–**i**) immunostained for CD31 (green) and APJ (red). At E12.5 and E13.5, the APJ-negative (immature) ECs migrated at the forefront of the sprouting subepicardial coronary vessels (arrows in **a**–**c** area 2, **d**–**f** area 4) and preceded the appearance of the APJ-positive (mature) ECs (arrowheads). At E14.5, all of the subepicardial ECs were double-positive for CD31 and APJ (arrowheads in **g**–**i**). (**j**–**l**) Schematic illustration of the SV, RA and RV at E12.5 (**j**), E13.5 (**k**) and E14.5 (**l**). Blue line, APJ-positive ECs; black line, APJ-negative ECs. Scale bars, 200 μm (upper panels in **a**–**i**); 50 μm (insets). RA, right atrium; RV, right ventricle; SV, sinus venosus.

The APJ-negative ECs were also detected in the ventricles of wild-type embryos both at E12.5 (Fig. 7a–c, j, arrows in area 2) and E13.5 (Fig. 7d–f, k, arrows in area 4). However, at E14.5, the APJ-negative subepicardial ECs could not be detected at the forefront of the invading vessels in the wild-type ventricles (Fig. 7g–i, l). These data suggest that immature APJ-negative ECs in the ventricles may differentiate into APJ-positive ECs at E14.5 in response to myocardial Ang1 (Fig. 7l).

Consistent with the whole-mount immunostaining with anti-APJ antibody results (Fig. 2c,d,g,h), APJ-positive ECs were observed in the basal subepicardial layers of both the right and left ventricles of control embryos, but not in those of Ang1CKO embryos at E13.5 (Supplementary Fig. 8a–g). Taken together, these data suggest that the APJ-negative ECs migrate from the SV into the atrium and subsequently into the ventricle at the forefront of the invading vessels and precede the appearance of APJ-positive ECs. The emergence of APJ-positive ECs occurs one day later in the atria and ventricles and requires the presence of myocardial Ang1.

In addition, we examined the proliferation of the subepicardial CD31-positive ECs in the hearts of both control and Ang1CKO embryos. Since we observed the invasion of CD31-positive subepicardial ECs from atria into the basal region of the ventricles in both control and Ang1CKO embryos (Fig. 1e,f), we examined the proliferation of CD31-positive subepicardial ECs in the ventricles of control and Ang1CKO embryos. The number of subepicardial ECs double-positive for pHH3 and CD31 in the ventricles of Ang1CKO embryos was 44% smaller than that in control embryos (Supplementary Fig. 9a–c), suggesting that myocardial deletion of Ang1 led to the impaired proliferation of CD31-positive subepicardial ECs.

We also examined the expression levels of *VEGF-A* and *VEGFR2* mRNAs in the hearts. They were almost comparable between control and Ang1CKO embryos (Supplementary Fig. 9d,e), indicating that the CD31-positive APJ-negative immature ECs in the subepicardial region of Ang1CKO hearts might be recruited from SV partly by the action of myocardium-derived VEGF-A. These findings suggest that myocardial Ang1 contributes not only to the migration but also to the proliferation of subepicardial immature ECs.

### Ang1 promotes venous differentiation of the immature ECs

The finding that APJ-negative ECs of the SV expressed APJ one or two days after migrating into the myocardium in control mice, but not in Ang1CKO mice suggested that Ang1 might be critically involved in the venous differentiation of immature ECs in the heart. Therefore, we examined the effect of Ang1 on arterial–venous specification using the Flk1-positive endothelial progenitor cells derived from embryonic stem (ES) cells (Flk1^+^ cells)^28^,^31^. Since the ECs that invade the atrium from the SV do not express either the arterial marker EphrinB2 or the venous marker APJ, we reasoned that the Flk1^+^ cells, which are double-negative for these markers, would be suitable for analysing the effects of Ang1 on the arterial–venous specification of the ECs. Treatment with VEGF and 8-bromo-cyclic-AMP (cAMP) upregulated the expression of the arterial marker EphrinB2 in Flk1^+^ cells, as reported previously (Supplementary Fig. 10a,b)^31^. Next, we examined the additive effects of treatment with cartilage oligomeric matrix protein (COMP)-Ang1, a potent variant of Ang1 (ref. 32)^32^, VEGF and cAMP on Flk1^+^ cells. Interestingly, treatment with COMP-Ang1 inhibited the induction of EphrinB2 by VEGF and cAMP (Supplementary Fig. 10c). qRT–PCR analysis also demonstrated that co-treatment with COMP-Ang1 counteracted the upregulation of *Efnb2* mRNA by VEGF and cAMP (Supplementary Fig. 10d).

**Figure 8:**
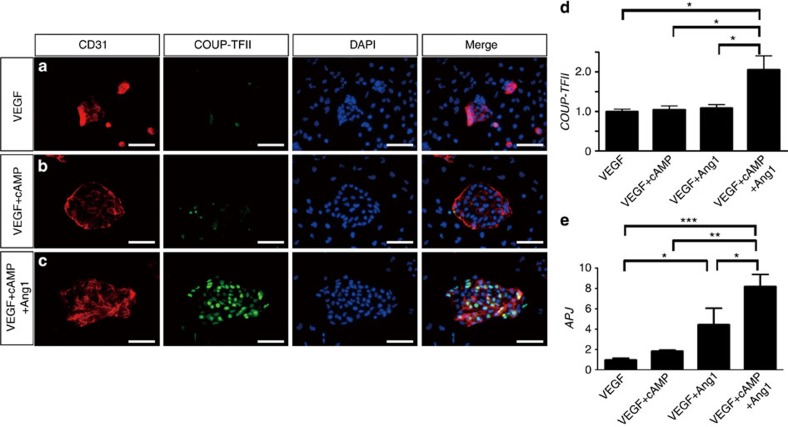
Ang1 enhances venous differentiation of Flk1^+^ immature endothelial progenitor cells synergistically with VEGF. (**a**–**c**) The venous marker protein COUP-TFII was upregulated in vascular progenitor Flk1^+^ cells by the addition of COMP-Ang1 to VEGF and cAMP. Flk1^+^ cells were immunostained with anti-CD31 antibody (red) and anti-COUP-TFII antibody (green). Nuclei were stained with DAPI (blue). (**d**–**e**) Quantitative expression analysis of the venous marker genes *COUP-TFII* and *APJ* in the Flk1^+^ cells (normalized to *GAPDH* mRNA; *n*=3). (**d**) The expression of *COUP-TFII* mRNA was increased exclusively by the combined treatment with VEGF, cAMP and COMP-Ang1. (**e**) The expression of *APJ* mRNA in the Flk1^+^ cells was significantly upregulated by stimulation with VEGF and Ang1 compared with treatment with VEGF alone. *APJ* mRNA was further upregulated by the addition of COMP-Ang1 to VEGF and cAMP. The results were expressed as relative intensity over cells treated with VEGF. Scale bars, 50 μm. Values are shown as means±s.e.m. for three separate experiments. One-way analysis of variance was used to compare differences. *P<0.05, **P<0.01, ***P<0.001 for the indicated groups.

In contrast, immunocytochemical analysis revealed that the transcription factor COUP-TFII, which is postulated to have a key role in venous differentiation^24^,^25^, was significantly upregulated in Flk1^+^ cells when stimulated with VEGF, cAMP and COMP-Ang1 (Fig. 8a–c). Consistent with these findings, qRT–PCR analysis revealed that co-stimulation with VEGF, cAMP and COMP-Ang1 upregulated the expression level of *COUP-TFII* in Flk1^+^ cells, although neither the co-stimulation with VEGF and cAMP nor that with VEGF and Ang1 upregulated the expression level of *COUP-TFII* (Fig. 8d).

Interestingly, co-stimulation with VEGF and COMP-Ang1 did upregulate the expression level of *APJ* mRNA in Flk1^+^ cells, whereas VEGF or co-stimulation with VEGF and cAMP did not significantly affect the expression level of *APJ*, indicating that APJ upregulation may be a prerequisite for the induction of COUP-TFII in Flk1^+^ cells (Fig. 8e).

In addition, we also examined the requirement of Tie2 receptor for the effect of Ang1 on the venous differentiation of Flk1-positive endothelial progenitor cells by siRNA-mediated knockdown of Tie2. We first confirmed that the expression level of Tie2 protein was significantly reduced by approximately 80% in the immature Flk1-positive endothelial progenitor cells (Supplementary Fig. 11a). We found that siRNA-mediated knockdown of Tie2 significantly blunted Ang1-dependent upregulation of *COUP-TFII* and *APJ* mRNAs (Supplementary Fig. 11b,c). These findings suggest that Tie2 activation is required for the Ang1-dependent venous differentiation of the immature Flk1-positive endothelial progenitor cells. Collectively, these data suggest that Ang1/Tie2 signalling promotes the venous differentiation of immature vascular progenitor Flk1^+^ cells via the upregulation of COUP-TFII and APJ. Since the growth factor(s) responsible for regulating COUP-TFII have not been identified to date, Ang1 might be a promising candidate factor for promoting the venous differentiation of immature ECs in the developing heart.

## Discussion

**Figure 9:**
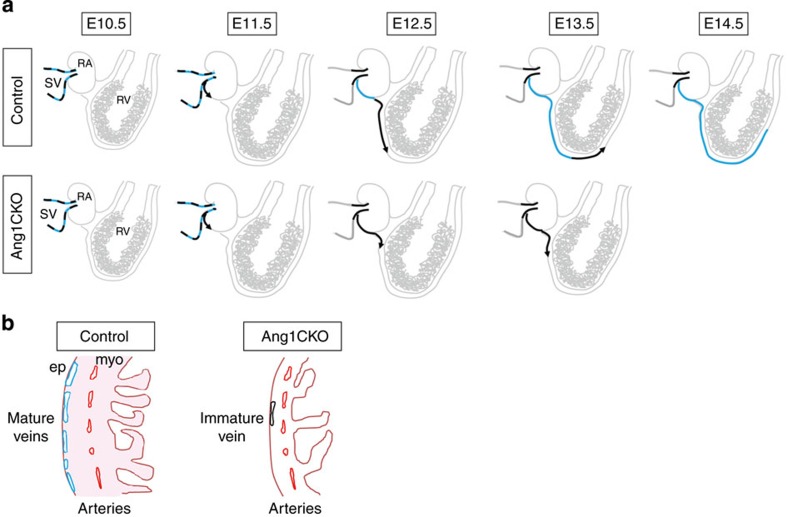
Role of Ang1 on coronary vessel formation. (**a**) Working model of coronary vein formation. The upper panel shows a schematic illustration of coronary vein formation in wild-type mice. The ECs in the SV at E10.5 consist of APJ-positive mature ECs (blue) and APJ-negative immature ECs (black). The APJ-negative ECs sprout off from the SV into the RA at E11.5. While the APJ-negative ECs migrate from the RA into the RV at E12.5, the APJ-negative ECs in the RA undergo venous differentiation in response to the action of Ang1 secreted from the myocardium, and differentiate into APJ-positive mature venous ECs. At E13.5, the APJ-negative ECs continue to migrate ahead of the APJ-positive ECs, which have emerged on the surface of the RV. At E14.5, all of the subepicardial CD31-positive cells have differentiated into mature venous APJ-positive ECs. In Ang1CKO mice, the migration, the proliferation and the venous specification of the APJ-negative ECs are impaired, resulting in defective coronary vein formation (lower panels). (**b**) Schematic diagram of coronary vessel formation in Ang1CKO embryo. Cardiomyocyte-specific deletion of Ang1 disturbs coronary vein formation, but does not impair coronary artery formation. Blue: APJ-positive ECs, Black: APJ-negative ECs. ep, epicardium; myo, myocardium; RA, right atrium; RV, right ventricle; SV, sinus venosus.

These findings demonstrate that Ang1 is essential for coronary vein formation in developing heart (Fig. 9a,b). Ang1CKO mice displayed significant defects in the migration of the APJ-negative immature ECs from the SV into the myocardium, the proliferation and the venous differentiation of the immature ECs. Together, Ang1CKO mice exhibited defective formation of subepicardial coronary veins. In addition, Ang1 in combination with VEGF and cAMP induced the venous differentiation of Flk1^+^ vascular progenitor cells, whereas the combination of VEGF and cAMP promoted arterial differentiation of the Flk1^+^ vascular progenitor cells. To our knowledge, this is the first report describing the growth factor responsible for the venous differentiation of immature ECs.

The origin of coronary ECs has been a long-lasting question. Ang1CKO mice displayed specific defects in the development of APJ-, EphB4-, COUP-TFII-positive coronary veins (Fig. 2); however, they showed no significant defects in coronary artery formation as evaluated by either coronary arteriogram with ink injection or detection of EphrinB2-lacZ-positive coronary arteries (Fig. 3). Wu *et al.*^11^ recently reported that both cardiomyocyte-specific VEGF-A KO mice and endocardium-specific VEGFR2 KO mice exhibit specific defects in coronary artery formation, but not in coronary vein formation. They reported that the ECs of the ventricular endocardium, but not those of the SV, generate the endothelium of coronary arteries. Their findings complement our findings obtained with Ang1CKO mice, suggesting that the formation of coronary arteries and veins might be distinctly regulated by VEGF-A/VEGFR2 signalling and Ang1/Tie2 signalling, respectively.

On the other hand, Red-Horse *et al.*^9^ reported that the ECs of the SV are the common originators of both coronary arteries and veins, using clonal analysis, crossing *VE-Cadherin-Cre(ER)T2* mice with either *Rosa-lacZ* or multicolour *Cre* recombination reporters, and organ culture experiments combining the SV and atrium from *Apelin-nLacZ*-reporter mice with ventricles from wild-type mice. We also performed organ culture experiments combining the SV and atrium from *Tie2-lacZ* reporter mice with the ventricles and epicardium from either wild-type or Ang1CKO mice. These experiments suggested that Ang1 derived from the ventricular myocardium might attract the Tie2-positive ECs from the SV towards the ventricular myocardium presumably via promotion of migration and proliferation of the immature ECs (Fig. 5). Since we could not address the arterial–venous identity of the Tie2-positive ECs migrating into the wild-type ventricles due to the loss of the antigenicity in the organ culture samples, we cannot exclude the possibility that the ECs of SV might partly contribute to the formation of coronary arteries independent of the action of myocardial Ang1. So, the issues on the origin of the coronary arteries should be clarified in the future.

Katz *et al.*^10^ recently reported that the Semaphorin3D/Scleraxis lineage-traced proepicardial cells, which migrate via SV endothelium into the myocardium and/or transiently contribute to the endocardium, differentiate into the coronary ECs. This finding indicates that the proepicardial cells as well as the SV ECs might be one candidate source for coronary vein ECs. Since there have been no appropriate reporter mice to specifically trace the lineage of the SV ECs to date, we should determine the origins of the coronary veins through identifying the marker genes specifically expressed in the ECs of the SV in the future.

Ang1 is reported to enhance the migration of the vascular ECs in cooperation with VEGF^33^. Consistent with this finding, Ang1CKO mice showed impaired migration of subepicardial immature APJ-negative ECs compared with control mice (Fig. 1 and Supplementary Fig. 2). Since the coronary artery formation was almost intact in Ang1CKO mice (Fig. 3), the myocardium-derived VEGF-dependent signalling and migration of ECs appeared to be preserved in these mice. These data indicate that Ang1 might regulate the migration of immature ECs from the SV into the myocardium in cooperation with VEGF derived from the myocardium.

On the other hand, we also found that Ang1CKO embryos showed impaired proliferation of subepicardial ECs compared with control embryos (Supplementary Fig. 9a–c). Previous studies revealed that Ang1 exerts opposing effects such as proliferation and stabilization of the cell–cell contact of the ECs depending on the cellular contexts^34^,^35^. While Ang1 can bridge Tie2 at cell–cell contacts and mediates *trans*-association of Tie2 in the presence of cell–cell contacts, extracellular matrix-bound Ang1 locates Tie2 at cell–substratum contacts in isolated cells^34^. Of note, Tie2 activated at cell–cell or cell–substratum contacts leads to preferential activation of Akt and Erk, respectively^34^,^35^, suggesting that Ang1 can evoke distinct cellular responses in the ECs according to the cellular environment. Taken together, myocardial Ang1 might contribute to the formation of coronary veins by promoting both proliferation and migration of the immature ECs derived from SV.

The ECs of the SV were found to be heterogeneous, consisting of both APJ-positive and -negative cells. Among the ECs in the SV, only the APJ-negative ECs were found to migrate into the atrium and ventricles. Furthermore, all of the atrial subepicardial ECs were APJ-positive following 1 day of exposure to Ang1 expressed in the atrial myocardium (Fig. 6). Thus, the APJ-positive ECs appeared to be produced through differentiation of the APJ-negative ECs by the action of myocardial Ang1.

Our findings indicate that myocardial Ang1 has a critical role in coronary vein formation by mediating the migration, proliferation and venous differentiation of immature ECs in the developing hearts. Elucidation of the molecular mechanisms underlying Ang1-mediated venous differentiation will provide insight into heart disease and tissue regeneration.

## Methods

### Animals

The targeting vector for creating *Ang1*^*flox*^ allele was constructed by inserting *loxP/PGK-Neo-pA/loxP* into exon 1 of the genomic *Ang1* locus^17^. The targeting vector was introduced into TT2 embryonic stem cells. The targeted embryonic stem cell clones were injected into CD-1 8-cell stage embryos, and the resultant male chimera mice were mated with C57BL/6 females to establish germ line transmission. *Ang1*^*flox(neo+)*^ mice were maintained in CBA/C57BL/6/CD-1 mixed background. The neomycin cassette in *Ang1*^*flox(neo+)*^ allele was excised through crossing with *CAG-FLPe* transgenic mice in C57BL/6 background (B6-Tg(*CAG-FLPe*)36)^36^. To create Ang1CKO mice, we crossed *Ang1*^*flox/flox*^ mice with *α-MHC-Cre* transgenic mice in a C57BL/6 background^16^,^17^. *Tie2-lacZ* transgenic (FVB/N-Tg(*TIE2-lacZ*)182Sato/J) mice were purchased from the Jackson Laboratory^30^. The *EphB4 tau-lacZ* knockin mice and *EphrinB2 tau-lacZ* knockin mice were used for determination of the arterial–venous lineages of the coronary ECs^23^,^27^. Enhanced green fluorescent protein (EGFP) reporter mice (CAG-CAT-EGFP) were obtained from J. Miyazaki, Osaka University^37^. The *Ang1-mCherry* gene construct was generated from a BAC clone (RP23-5J11) containing a 144-kb genomic fragment spanning the region upstream of the *Ang1* locus. The targeting vector used to modify BAC RP23-5J11 was designed to insert the *mCherry* sequence into the first coding exon of the *Ang1* gene. The 5′ and 3′ homology sequence, the *mCherry* gene, and the fragment including the poly A sequence were cloned into PL453, which contained the neomycin-resistance cassette flanked by Frt sites.

All animals were maintained in a virus-free facility on a 12-h light/12-h dark cycle and fed a standard mouse diet. All experiments were carried out under the guidelines of the Osaka University Committee for animal and recombinant DNA experiments and were approved by the Osaka University Institutional Review Board.

### Genotyping of the animals

The PCR primers used for genotyping were as follows: Ang1 flox: 5′-CCGGATTCAACATGGGCAATGTGCC-3′, 5′-CAGTCAAAATGCCTAAGATAAAC-3′; Cre: 5′-ACATGTTCAGGGATCGCCAG-3′, 5′-TAACCAGTGAAACAGCATTGC-3′; lacZ: 5′-CAGACGATGGTGCAGGATAT-3′, 5′-ATACAGCGCGTCGTGATTAG-3′; Ang1-mCherry: 5′-AGGACGGCGAGTTCATCTAC-3′, 5′-TGGTGTAGTCCTCGTTGTGG-3′; EGFP: 5′-AGCAAGGGCGAGGAGCTGTT-3′, 5′-GTAGGTCAGGGTGGTCACGA-3′.

### Detection of β-galactosidase activity in embryonic tissues

Embryonic tissues were fixed in 0.2% glutaraldehyde in phosphate-buffered saline (PBS) containing 5 mM EGTA and 2 mM MgCl_2_ at 4 °C for 4 h, washed in washing buffer (2 mM MgCl_2_, 0.02% NP-40, 0.1% sodium deoxycholate in PBS) for 30 min three times, cryoprotected with 30% sucrose, and frozen in OCT compound (Tissue Tek) and cryosectioned. Samples were stained in X-gal staining buffer (5 mM potassium ferricyanide, 5 mM potassium ferrocyanide, 1 mg/ml X-gal in washing buffer) at 37 °C.

### Histological analysis

Dissected embryonic hearts were fixed in 4% paraformaldehyde (PFA)/PBS overnight at 4 °C, embedded in paraffin and sectioned at 7 μm thickness. Hematoxylin/eosin staining was performed according to standard procedures on the paraffin sections^16^.

### Immunohistochemical analysis

The following antibodies were used: anti-CD31 (550274) and anti-VEGFR2 (550549) (BD Pharmingen), anti-Tie2 (sc-324, Santa Cruz), anti-beta-galactosidase (NB100-2045, Novus Biologicals), anti-DsRed (632496, Clontech), anti–phospho–Histone H3 (Ser10) (06-570, Merck Millipore). The whole-mount immunohistochemistry of mouse embryos using anti-CD31 monoclonal (1:100) and anti-APJ antibodies (1:100) was performed^38^,^39^. Embryonic hearts were dissected and fixed in 4% PFA/PBS overnight at 4 °C. Samples were washed in PBS, dehydrated in absolute methanol (MeOH) and stored at −20 °C until antibody staining. Embryonic hearts were incubated in Dent’s bleach (MeOH: dimethyl sulfoxide: 30%H_2_O_2_, 4:1:1) for 3 h at room temperature and washed with a series of descending MeOH/PBST (PBS+0.1% Triton-X) concentrations (70%MeOH, 50%MeOH, PBST). Samples were blocked in 2% skimmed milk for 1 h and incubated with primary antibodies overnight at 4 °C. Next day samples were washed three times for 1 h with PBST and incubated with secondary antibodies overnight at 4 °C. Finally samples were washed three times for 1 h with PBST and applied with DAB (Invitrogen) at room temperature. When necessary, 7 μm sections were cut from paraffin-embedded whole-mounts.

For frozen section immunohistochemistry, embryos or isolated embryonic hearts were fixed for 4 h to overnight in 4% PFA/PBS. Fixed embryos or hearts were soaked in a 15–30% sucrose gradient before being embedded in OCT (Tissue Tek) for frozen sections and cut by cryosectioning (10 μm). Sections were rehydrated in PBS, incubated in blocking solution containing either 5% normal goat serum, 1% bovine serum albumin and 2% skim milk or 10% normal goat serum in PBST for 1 h and then incubated with primary antibodies (CD31 1:300, Tie2 1:200, APJ 1:300, VEGFR2 1:100, Beta-galactosidase 1:4000, DsRed 1:1000, phospho Historne H3 1:100) in Can Get Signal immunostain (TOYOBO, NKB-601) overnight at 4 °C. Sections were then washed in PBST and incubated with fluorescent-conjugated (Invitrogen; Alexa Fluor 488 and 546) or horseradish peroxidase (HRP)-coupled (Cell Signaling Technology (7074), 1:200) secondary antibodies for 30 min to 1 h at room temperature. Images were acquired with a microscope (Keyence, BZ-9000).

### Whole-mount *in situ* hybridization

Embryos were fixed in 4% PFA/PBS overnight at 4 °C and stored in 100% MeOH at −20 °C until hybridization. The Ang1 antisense probe was generated from mouse *Ang1* cDNA^18^. Whole-mount *in situ* hybridization was performed at 70 °C for 18 h for each sample^40^. The detection was performed with alkaline phosphatase-coupled anti-digoxygenin antibody (Boehringer Ingerheim) overnight at 4 °C. After washing, the chromogenic reaction was performed with NBT–BCIP substrate (Promega). Photographs were captured with a stereomicroscope (Olympus, SZX12).

### *In situ* hybridization on frozen sections

Embryos were fixed overnight in 4% PFA/PBS. Fixed embryos were soaked in a 15–30% sucrose gradient before being embedded in OCT (Tissue Tek) for frozen sections (10 μm). Sections were rehydrated in PBS. A 994 bp long *in situ* probe was generated of the mouse *COUP-TFII* gene (Forward: CGGAATTCTCAACTGCCACTCGTACCT, Reverse: CCACTAGTGCTTTCCACATGGGCTACAT). *In situ* hybridization was performed in a similar method to whole-mount *in situ* hybridization^40^. Hybridized DIG-RNA probes were detected with alkaline phosphatase-coupled anti-digoxygenin antibody overnight at 4 °C. Photographs were captured with BZ-9000 (KEYENCE).

### Coronary arteriogram of murine embryos

Hearts were resected from embryos and placed in heparinized PBS. Ink (Kiwa-guro; Sailor) was injected in a retrograde fashion from the ascending aorta using a glass micropipette and fixed in 4% PFA/PBS^26^. Photographs were captured using a stereomicroscope (Olympus, SZX12).

### Quantitative real-time RT–PCR

Quantitative real-time RT–PCR was carried out using the QuantiFast SYBRGreen RT–PCR kit (Qiagen)^39^. For each reaction, 80 ng of total RNA was transcribed for 10 min at 50 °C followed by a denaturing step at 95 °C for 5 min and 40 cycles of 10 s at 95 °C and 30 s at 60 °C. Fluorescence data were collected and analysed using ABI PRISM 7900HT. The primers used for amplification of total RNA from murine hearts were as follows: *GAPDH*: 5′-TCTCCACACCTATGGTGCAA-3′, 5′-CAAGAAACAGGGGAGCTGAG-3′; *Ang1* 5′-GCAGCCATAGCAATGCCAGAGGT-3′, 5′-TCCCATGGCAACTCACAAAACTCC-3′; *Efnb2*: 5′-TGTTGGGGACTTTTGATGGT-3′: 5′-GTCCACTTTGGGGCAAATAA-3′; *Ephb4*: 5′-CTGGATGGAGAACCCCTACA-3′, 5′-CCAGGTAGAAGCCAGCTTTG-3′; *COUP-TFII*: 5′-GCAAGTGGAGAAGCTCAAGG-3′, 5′-TTCCAAAGCACACTGGGACT-3′; *Notch1*: 5′-TGTTGTGCTCCTGAAGAACG-3′, 5′-TCCATGTGATCCGTGATGTC-3′; *Dll4*: 5′-TGCCTGGGAAGTATCCTCAC-3′, 5′-GTGGCAATCACACACTCGTT-3′; *Acvrl1*: 5′-CCAATGACCCCAGTTTTGAG-3′, 5′-TTGGGGTACCAGCACTCTCT-3′; *Hes1*: 5′-ATCATGGAGAAGAGGCGAAG-3′, 5′-CGGAGGTGCTTCACAGTCAT-3′; *Nrp1*: 5′-CCGGAACCCTACCAGAGAAT-3′, 5′-AAGGTGCAATCTTCCCACAG-3′; *APJ*: 5′-CAGTCTGAATGCGACTACGC-3′, 5′-CCATGACAGGCACAGCTAGA-3′.

### Organ cultures

Heart cultures were carried out according to the previous report^9^. In brief, embryonic hearts were dissected from wild-type, *Tie2-lacZ* or Ang1CKO embryos, and the atria and attached sinus venosus (SV/A) were then dissected from the ventricles. The SV/A tissues from the *Tie2-lacZ* embryos were placed adjacent to the ventricles of either wild-type or Ang1CKO embryos at the position where the original SV/A was removed. The explants were cultured dorsal side up at the air–liquid interface on 8-mm Millicell Cell Culture Insert Filters (Merck Millipore). Cultures were maintained at 37 °C and 5% CO_2_ in DMEM with 2 μg ml^−1^ heparin, 100 U ml^−1^ penicillin, 100 μg ml^−1^ streptomycin, 2 mM L-glutamine and 10% fetal bovine serum. After 72 h, explants were fixed with 4% PFA/PBS and subjected to whole-mount X-gal staining. Some stained explants were embedded in paraffin and then sectioned.

### Cell culture and differentiation of embryonic stem cells

ES cell lines EStTA-ROSA, and various EStTA derivatives were maintained with Glasgow’s MEM (GMEM; Invitrogen) containing 1 × 10^−4^ M 2-mercaptoethanol (Invitrogen), 10% knockout serum replacement (KSR; Invitrogen), 1% fetal calf serum (FCS; SAFC Biosciences), 1 mM sodium pyruvate (Sigma), 1% non-essential amino acids solution (Invitrogen) and 2 × 10^3^ U ml^−1^ leukaemia inhibitory factor (Merck Millipore). Differentiation was induced in these ES cell lines using differentiation medium (DM) consisting of minimum essential medium alpha (Invitrogen) supplemented with 10% FCS (Invitrogen) and 5 × 10^−5^ M 2-mercaptoethanol^31^,^41^. In brief, undifferentiated ES cells were cultured on gelatin-coated dishes without leukaemia inhibitory factor at a density of 0.75–10^3^ cells per cm^2^ for 4.5 days. Cultured cells were harvested and subjected to magnetic cell sorting (MACS) purification. Purified Flk1^+^ cells were then plated onto gelatin-coated dishes at a density of 0.75–10^4^ cells per cm^2^ in DM. After 3 days, induced ECs were examined by immunocytochemistry and FACS analysis. Human VEGF_165_ (WAKO, 50 μg ml^−1^) and 8-bromoadenosine-3′, 5′-cyclic monophosphate sodium salt (8-bromo-cAMP; Nacalai, 0.5 mM) were added to the Flk1^+^ cell culture. COMP-Ang1 was added to the Flk1^+^ cell culture at the concentration of 100 ng ml^−1^ (ref. 32).

Stealth siRNAs targeting murine *Tie2* gene were purchased from Invitrogen (MSS211290, MSS211291, MSS278161). Cells were transfected with mixed three siRNAs (total 50 nM) 12 h before Flk1^+^ cell purification using Lipofectamine RNAiMAX reagent (Invitrogen) according to the manufacturer’s instructions. Furthermore, Flk1^+^ cells were plated and simultaneously transfected with mixed siRNAs (total 20 nM).

### Immunocytochemical analysis of Flk1^+^ cells

The cultured ECs were immunostained according to the previous report^41^. Briefly, for double-fluorescence staining of COUP-TFII and CD31, ECs were fixed with 4% PFA/PBS. Fixed culture slides were incubated with anti-COUP-TFII antibody (H7147, Perseus Proteomics) and anti-CD31 (550274, BD Pharmingen). Culture slides were then washed in PBST and incubated with fluorescent-conjugated secondary antibodies (Invitrogen; Alexa Fluor 488 and 546). For double-fluorescence staining of EphrinB2 and CD31, ECs were fixed with 5% dimethyl sulfoxide/MeOH. Fixed culture slides were incubated with EphB4-human immunoglobulin (Ig) Fc portion chimeric protein (EphB4-Fc; R&D Systems) followed by human IgG Fc peroxidase-conjugated goat IgG fraction (ICN Biomedicals). The TSA biotin system (PerkinElmer) was used to amplify the signal for EphB4-Fc staining. EphrinB2-positive cells were visualized with streptavidin/Alexa Fluor 488 conjugate (Invitrogen). CD31-positive cells were stained with PE-conjugated mAb for CD31.

### Statistical analysis

All data were expressed as means±s.e.m. Differences among multiple groups were compared by one-way analysis of variance. Student’s *t*-test was used to analyse differences between two groups. A value of *P*<0.05 was considered as statistically significant.

## Author contributions

Yo.A. performed most of experiments in mice, analysed data, wrote manuscripts; Y.N. designed this study, wrote manuscript, created mice through crossing, analysed data and supervised this study; T.Mat., K.Y., and J.K.Y. performed experiments using Flk1+ vascular progenitor cells and analysed data; Yu.A., K.N. and H.Ku. performed the experiments of murine coronary arteriogram; H.Ki., T.K.-H., K.I., T.Y., T.Mas., K.Y., K.H., M.S., H.Y. and T.Mi. performed the mice experiment; K.O., N.M. and N.T. contributed to the generation of mice; K.Y.-T., and Y.S. helped the design of this study; J.-S.P. and G.Y.K. generated COMP-angiopoietin-1; I.K. edited the manuscript and supervised this project generally.

## Additional information

**How to cite this article:** Arita, Y. *et al.* Myocardium-derived angiopoietin-1 is essential for coronary vein formation in the developing heart. *Nat. Commun.* 5:4552 doi: 10.1038/ncomms5552 (2014).

## Supplementary Material

Supplementary Information Supplementary Figures 1-11 and Supplementary Table 1
