# Supplementary material for: Myocardium-derived angiopoietin-1 is essential for coronary vein formation in the developing heart
Source: Nat Commun. 2014 Jul 29;5:4552. doi: 10.1038/ncomms5552 (PMC4124867; doi:10.1038/ncomms5552)
Supplement: Supplementary Information — Supplementary Figures 1-11 and Supplementary Table 1 [file ncomms5552-s1.pdf]

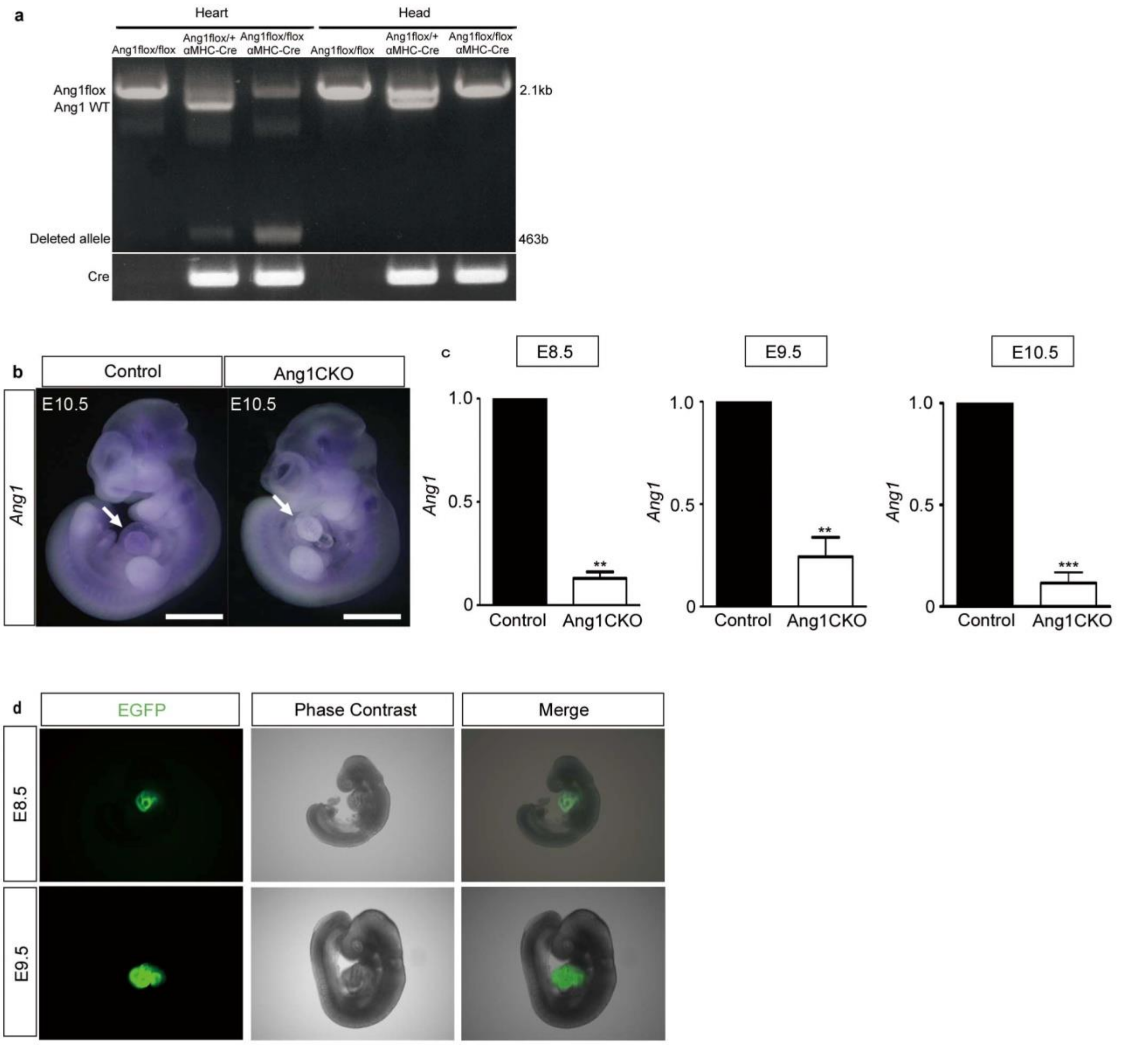

Supplementary Figure 1 | Generation of *Ang1*^{flox} and Ang1CKO mice

(**a**) Genomic PCR analysis of DNA samples from E11.5 demonstrated recombination of the *Ang1*^{flox} allele in the heart, but not in the head of *Ang1flox/flox* and *Ang1flox/+* embryos that possessed the *α-MHC-Cre* allele. (**b**) Whole-mount in situ hybridization at E10.5 of *Ang1* mRNA in control and Ang1CKO embryos. *Ang1* mRNA was successfully deleted in the heart of Ang1CKO embryos. (**c**) *Ang1* mRNA was successfully deleted in the heart of Ang1CKO embryos at E8.5, E9.5 and E10.5 as assessed by qRT-PCR. (**d**) α-MHC-Cre reporter analysis. α-MHC-Cre mice were bred with enhanced green fluorescent protein (EGFP) reporter mice (CAG-CAT-EGFP mice). The expression of EGFP in the hearts of offsprings which possessed both *α-MHC-Cre* and *CAG-CAT-EGFP* allele was observed at E8.5 and E9.5. Values are shown as means ± SEM for four separate experiments. Student's *t*-test was used to analyze differences. ***P*<0.01, ****P*<0.001 compared with control.

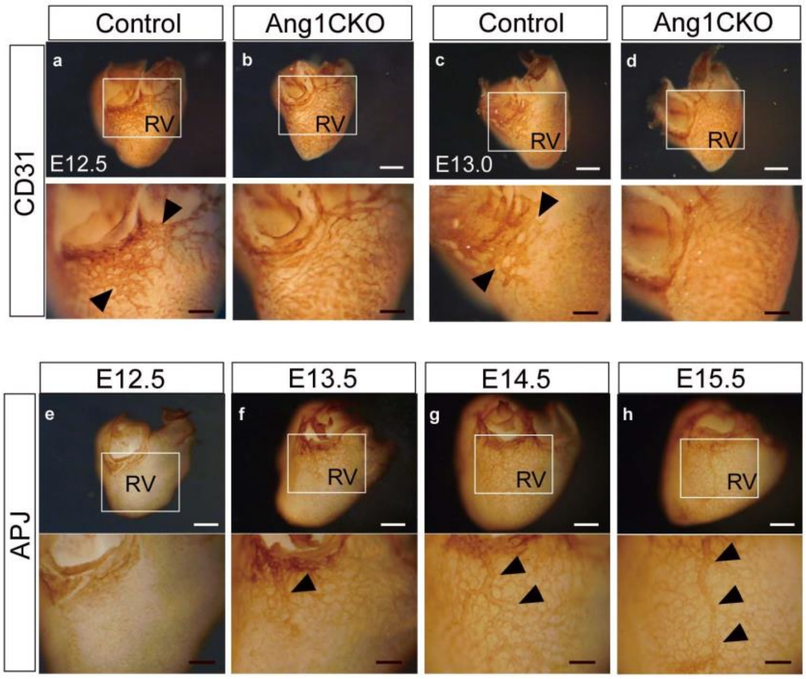

Supplementary Figure 2 | Ang1CKO embryos display defects in subepicardial coronary vein formation

(**a**-**d**) Whole-mount immunostaining of the hearts of control and Ang1CKO embryos with an anti-CD31 antibody at E12.5 (**a**, **b**) and E13.0 (**c**, **d**). Superficial CD31-positive coronary vessel formation (arrowheads in magnified image of inset) was observed in control, but not in Ang1CKO embryos. (**e**-**h**) Whole-mount immunostaining of embryonic wild-type hearts with an anti-APJ antibody. (**e**) APJ-positive vessels were not observed on the surface of ventricles at or before E12.5. (**f**-**h**) The APJ-positive coronary veins were observed on the surface of ventricles of wild-type embryos at and after E13.5 (arrowheads in magnified image of inset). Scale bars: 400 μm (upper panels in a-h); 100 μm (lower panels in a-h). RV, right ventricle.

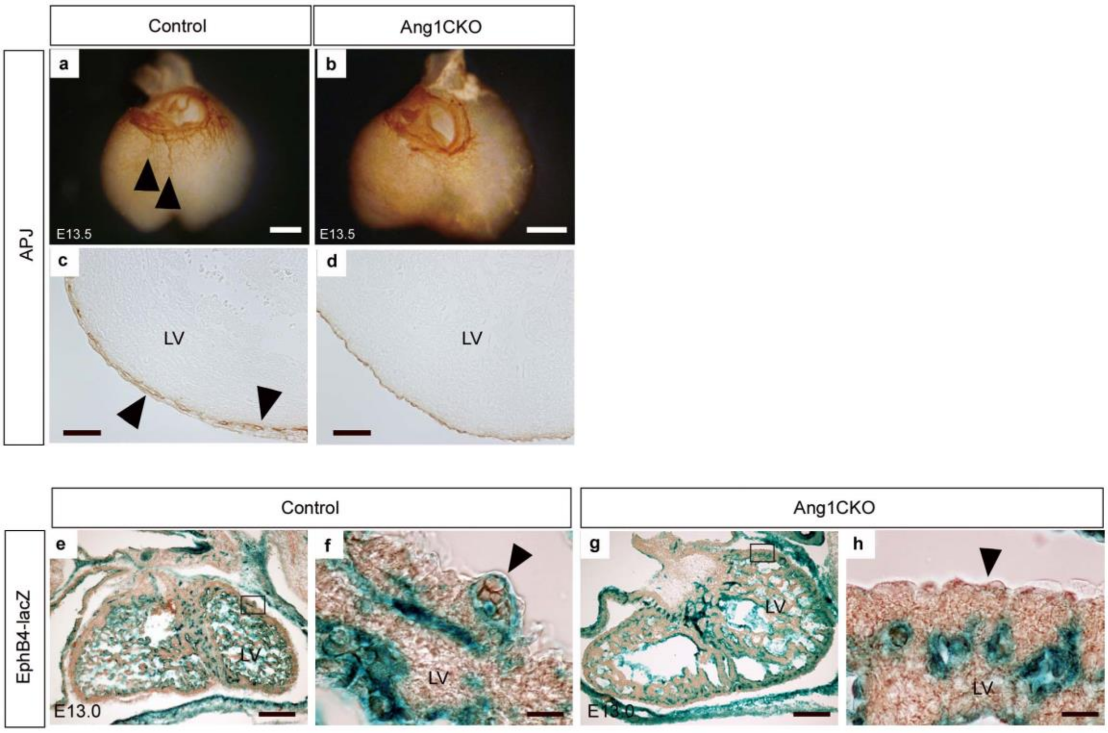

Supplementary Figure 3 | Myocardial Ang1 is essential for coronary vein formation

(**a-d**) Whole-mount immunostaining of embryonic hearts with anti-APJ antibody. APJ-positive coronary veins were observed on the surfaces of the LV of control embryos (**a**, arrowheads), but not on that of Ang1CKO embryos (**b**). Sectioned analyses of the whole-mount immunostained embryonic hearts revealed subepicardial APJ-positive coronary veins with vessel-like structures in the LV (**c**, arrowheads) of control, but not Ang1CKO embryos (**d**). These samples were the same samples with those shown in Fig.2c-d. (**e-h**) EphB4-lacZ-positive signals in the hearts of control and Ang1CKO embryos at E13.0. EphB4-lacZ-positive subepicardial coronary veins were observed on the surfaces of both the RV (Figure2, j) and the LV in control (**f**), but not observed in Ang1CKO embryos (**h**). EphB4-lacZ-positive signals were also detected in the endocardial endothelium in both control and Ang1CKO embryos. These samples were the same samples with those shown in Fig.2i-l. Scale bars; 200 μm in **a**, **b**; 50 μm in **c**, **d**; 300 μm in **e**, **g**; 50 μm in **f**, **h**. RV; right ventricle, LV; left ventricle.

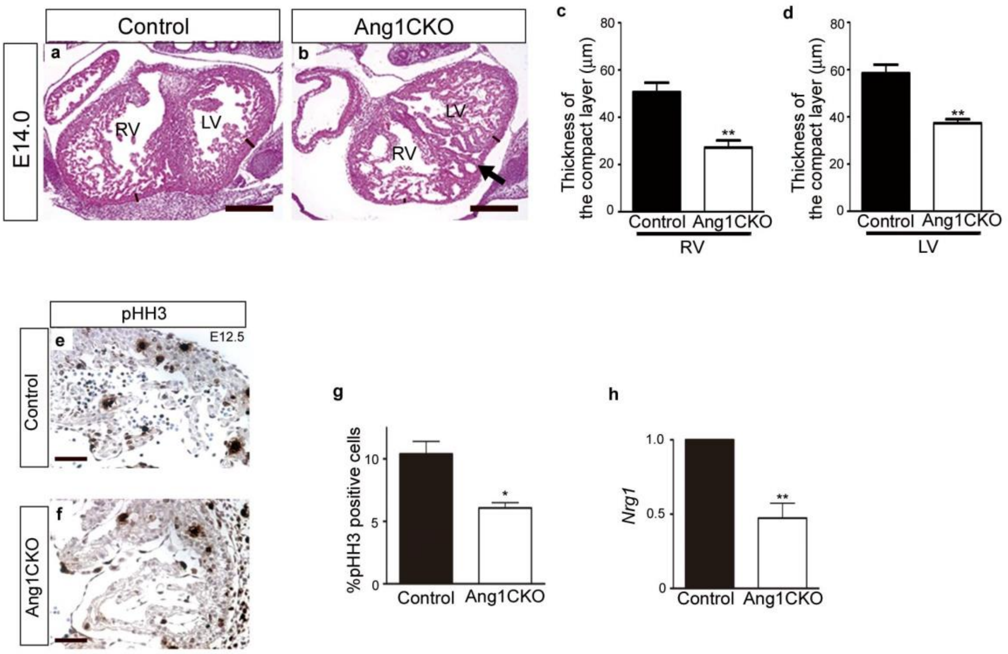

Supplementary Figure 4 | Ang1CKO embryos exhibit impaired development of both trabeculae and compact layers of the myocardium

(**a-b**) Representative images of HE staining of heart sections at E14.0. The black bars indicate the thickness of the compact layers of both RV and LV. The arrow indicates impaired interventricular septum in Ang1CKO embryos. (**c-d**) The thickness of the compact layer of both RV and LV in Ang1CKO embryos was significantly thinner than that in control embryos (n=3). (**e-g**) The proliferation of cardiomyocytes in the hearts at E12.5 assessed by immunostaining with anti-pHH3 antibody. The number of pHH3-positive myocardial cells was significantly decreased in Ang1CKO embryos compared with control embryos (**g**). (**h**) The expression level of *NRG1* mRNA was significantly decreased in the hearts of Ang1CKO embryos compared with those of control embryos at E11.5. Scale bars: 200 μm in **a**, **b**, 50 μm in **e, f**. Values are shown as means ± SEM for three separate experiments. RV; right ventricle, LV; left ventricle. Student's *t*-test was used to analyze differences. *P<0.05, **P<0.01 compared with control.

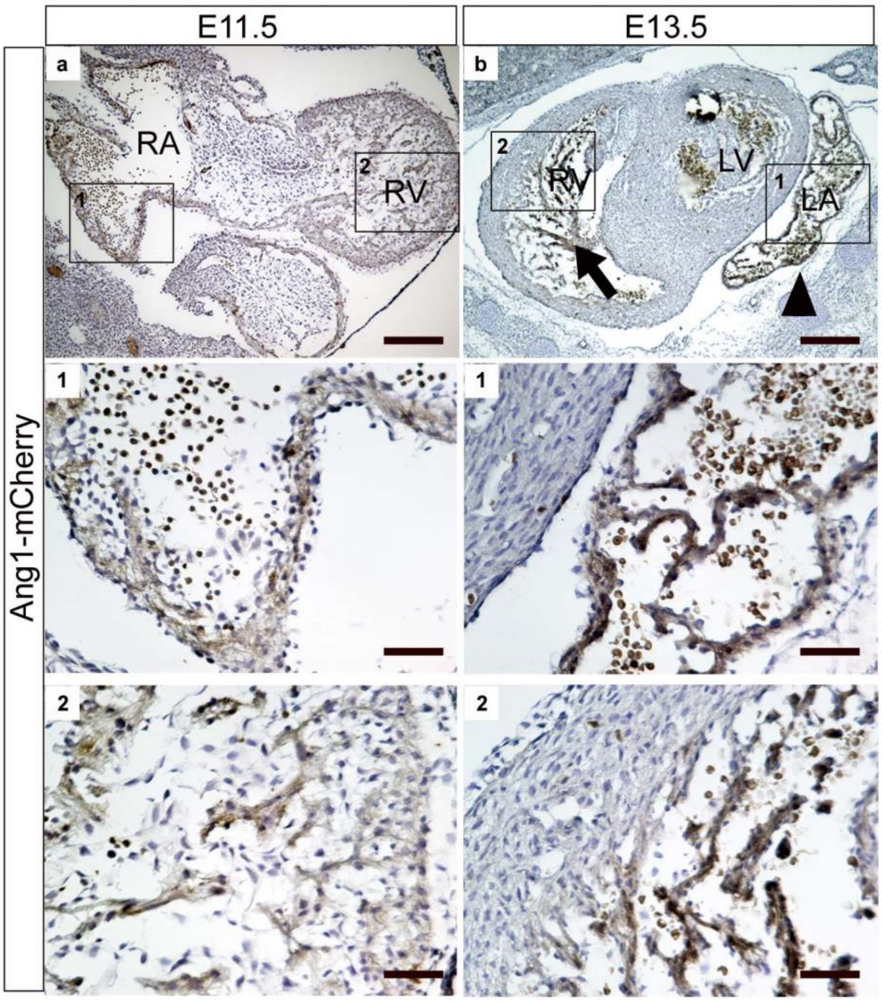

Supplementary Figure 5 | Expression patterns of Ang1 in the heart and the SV

(**a-b**) Expression patterns of Ang1 in the hearts of Ang1mCherry reporter mice at E11.5 (**a**) and E13.5 (**b**, arrow; ventricular trabeculae, arrowhead; LA). Scale bars: 200 μm in **a**, 250 μm in **b**, 50 μm in insets. RA, right atrium; RV, right ventricle; LV, left ventricle; LA left atrium.

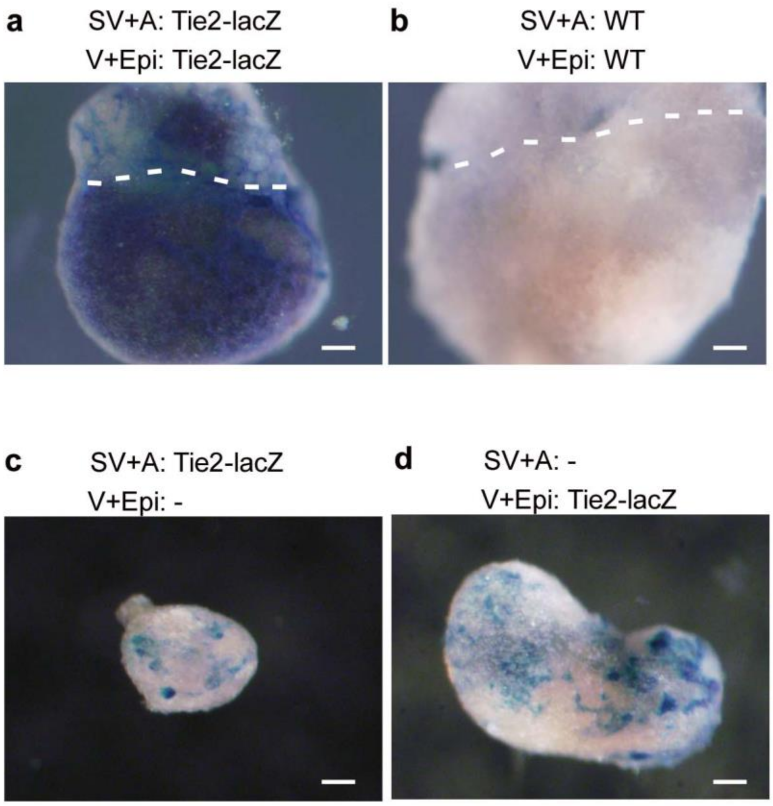

Supplementary Figure 6 | X-gal staining of the intact and dissected hearts from *Tie2-lacZ* transgenic embryos

(**a-b**) Intact or (**c-d**) dissected hearts from E10.5 *Tie2-lacZ* transgenic embryos (**a, c, d**) or wild type embryos (**b**) were cultured for 3 days and stained with X-gal. The intact and dissected hearts from *Tie2-lacZ* embryos were lacZ-positive because Tie2 was expressed throughout the endocardial endothelium. Scale bars; 100 μm. SV, sinus venosus; A, atrium; V, ventricle; Epi, epicardium.

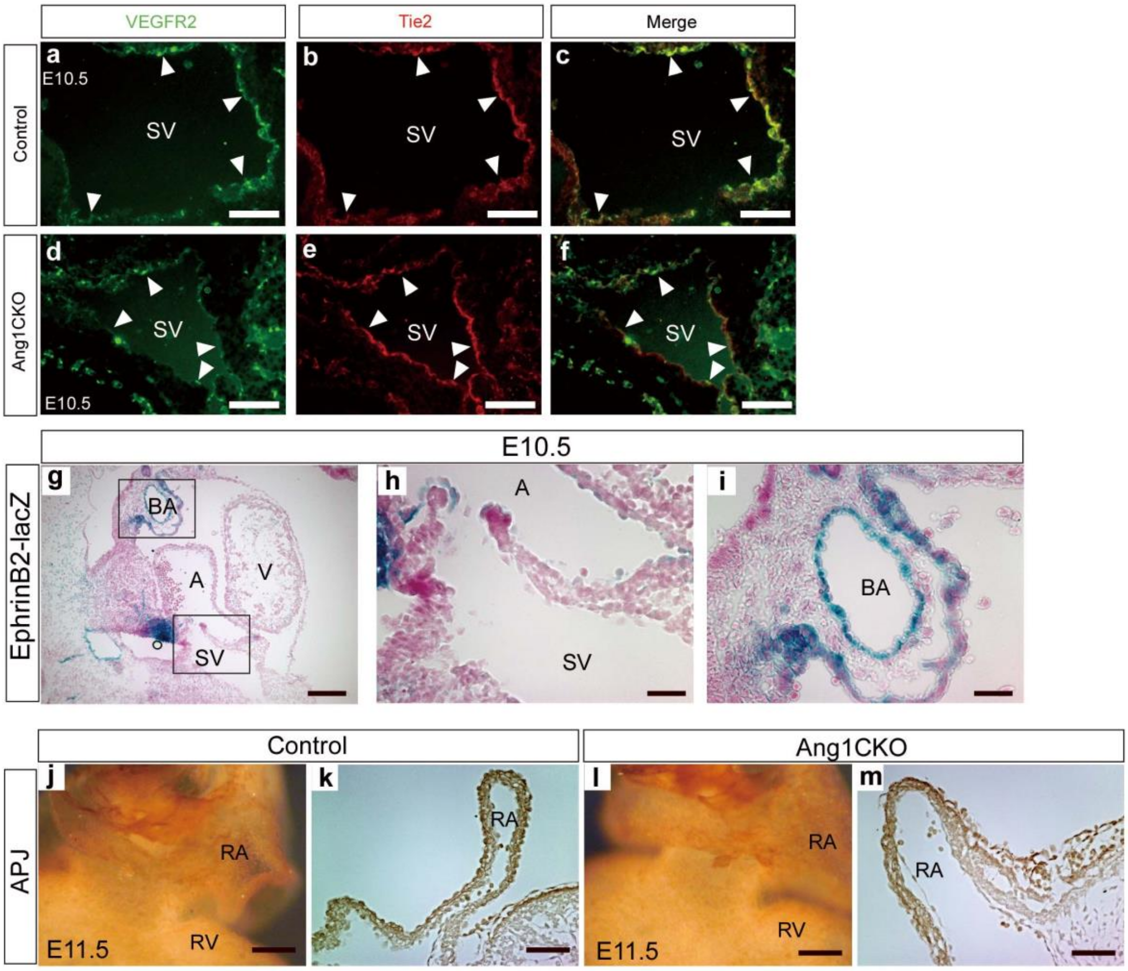

Supplementary Figure 7 | Expression of endothelial cell markers in the SV and atrium during embryogenesis

(**a-f**) Sagittal section through the sinus venosus (SV) of control (**a-c**) and Ang1CKO embryos (**d-f**) at E10.5 immunostained for VEGFR2 (green) and Tie2 (red). Both VEGFR2 and Tie2 were uniformly expressed in the ECs of the SV (arrowheads), and the expression patterns of VEGFR2 and Tie2 were similar in control and Ang1CKO embryos (arrowheads). (**g-i**) X-gal staining of *EphrinB2-lacZ* transgenic mice at E10.5. EphrinB2-positive signals were not observed in the SV, but observed in the branchial artery. (**j-m**) Whole-mount immunostaining of the hearts of control and Ang1CKO embryos at E11.5 with an anti-APJ antibody. APJ-positive coronary veins were not observed on the surface of the RA from either control or Ang1CKO embryos at E11.5 (**j, l**). Sectioned analysis of the whole-mount immunostained embryonic hearts demonstrated that the APJ-positive coronary veins with vessel-like structures were not found in the RA of either control or Ang1CKO embryos (**k, m**). Scale bars; 50 μm in **a-f**, **h-i**; 200 μm in **g**; 100 μm in **j, l**; 50 μm in **k, m**. RA, right atrium; SV, sinus venosus; BA, branchial artery.

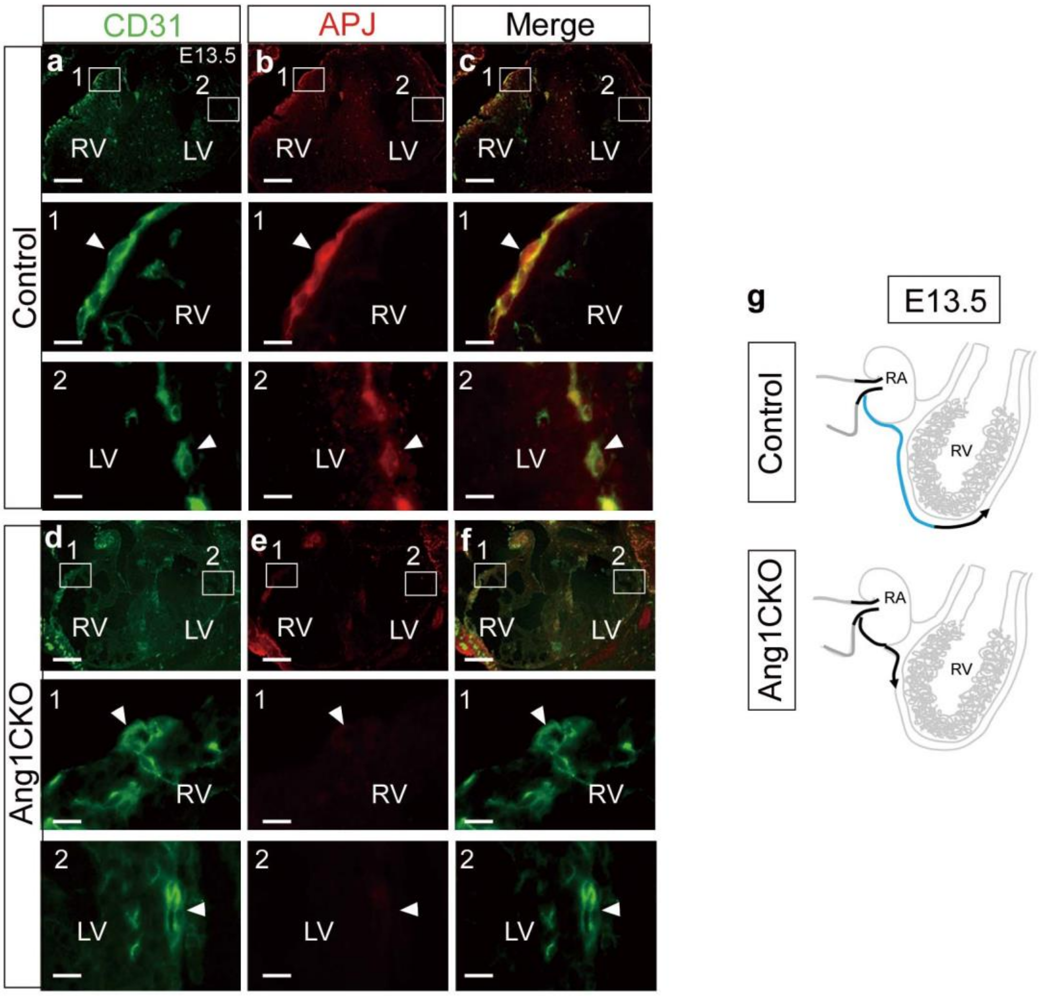

Supplementary Figure 8 | APJ is expressed in the subepicardial coronary ECs of control, but not Ang1CKO mice

(**a-f**) Coimmunostaining of CD31 and APJ, and merged image in the heart. At E13.5, APJ was detected in the superficial CD31-positive coronary ECs of wild-type control embryos (arrowheads in **a-c**), but not Ang1CKO embryos (arrowheads in **d-f**). (**g**) Schematic illustration of the SV, RA, and RV at E13.5. Blue line: APJ-positive ECs, Black line: APJ negative ECs. Scale bars: 200 μm (upper panels); 50 μm (insets). RV, right ventricle; LV, left ventricle.

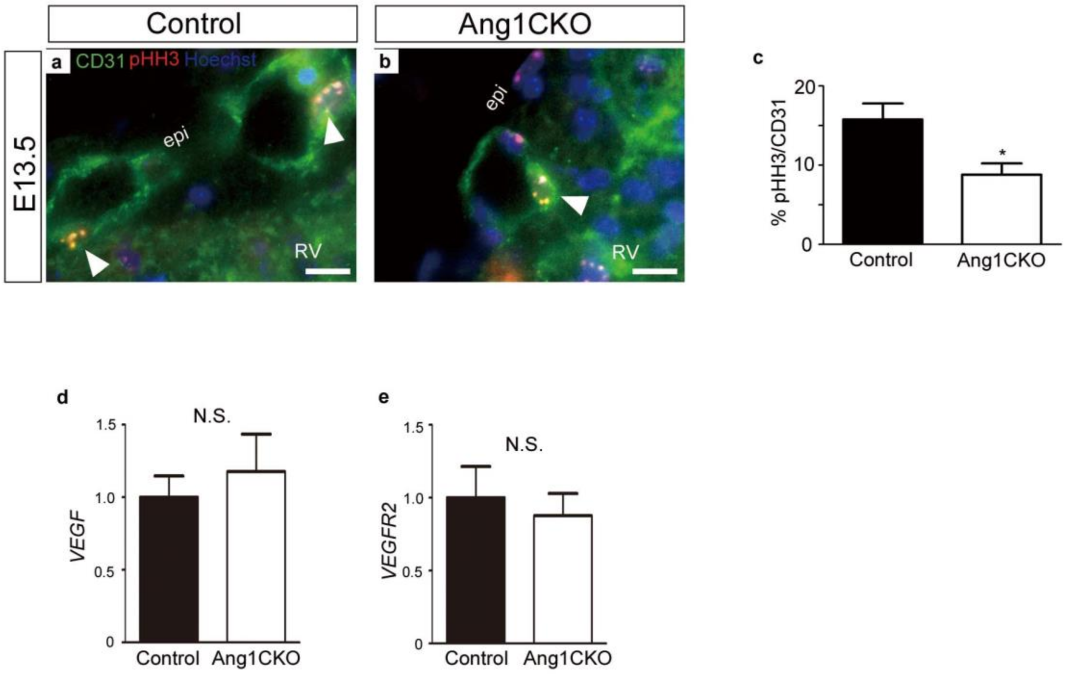

Supplementary Figure 9 | Ang1CKO embryos exhibited significantly decreased proliferation of subepicardial endothelial cells compared with control embryos.

(**a-c**) Coimmunostaining with anti-CD31 and anti-pHH3 antibodies in the heart at E13.5. The number of subepicardial endothelial cells doubly positive for CD31 and pHH3 was significantly reduced in the Ang1CKO embryos (arrowheads in **b**) compared with that in control embryos (arrowheads in **a**). The pHH3/CD31 ratio in the subepicardial coronary ECs was significantly decreased in Ang1CKO embryos compared with that in control embryos (**c**). (**d-e**) By qRT-PCR, the expression levels of *VEGF* and *VEGFR2* mRNAs were almost similar in the hearts of control and Ang1CKO embryos at E12.5-E13.0 (n=3). Scale bars: 10 μm in **a**,**b**. RV, right ventricle; epi, epicardium. Values are shown as means ± SEM for three separate experiments. Student's *t*-test was used to analyze differences. *$P<0.05$ compared with control. N.S.; not significant.

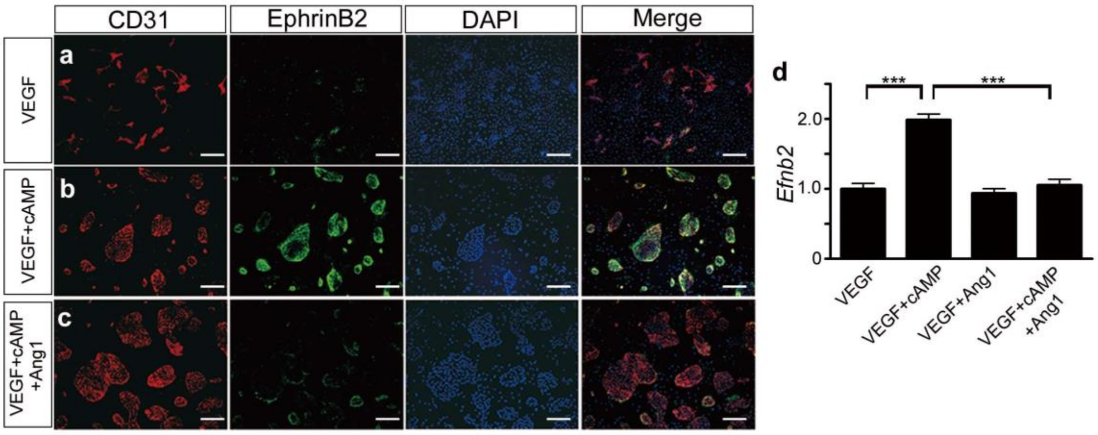

Supplementary Figure 10 | Ang1 inhibits arterial differentiation of Flk$^+$ immature endothelial progenitor cells

(**a-c**) The arterial marker protein EphrinB2 (green) was upregulated by VEGF and 8-bromo-cAMP (cAMP), but suppressed by addition of COMP-Ang1 to VEGF and cAMP in the vascular progenitor Flk1+ cells. ECs were immunostained with an anti-CD31 antibody (red). Nuclei were stained with DAPI (blue). (**d**) The expression level of *Efnb2* mRNA was upregulated by stimulation with VEGF and cAMP, but suppressed by addition of COMP-Ang1 to VEGF and cAMP in the vascular progenitor Flk1+ cells. Scale bars, 50 μm. The result was expressed as relative intensity over cells treated with VEGF. Values are shown as means ± SEM for three separate experiments. One-way ANOVA was used to compare differences. $^{***}P<0.001$ for the indicated groups.

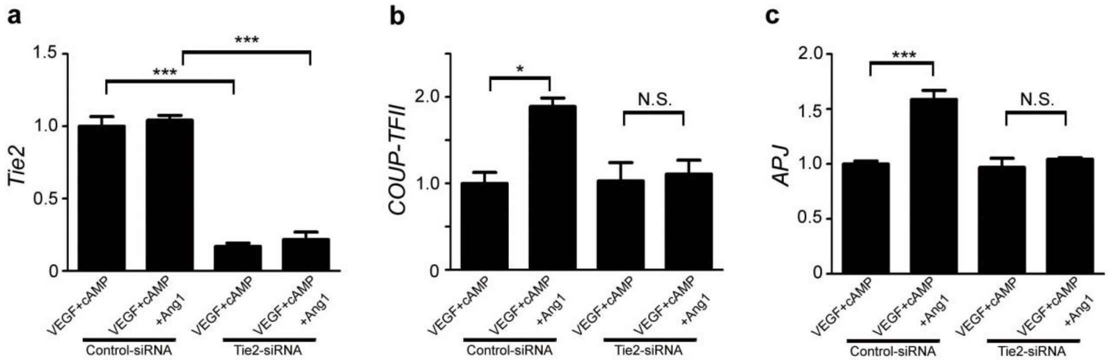

Supplementary Figure 11 | Ang1 promotes venous differentiation of Flk1^{+} immature endothelial progenitor cells through Tie2 receptor

(**a-c**) Quantitative expression analysis of the *Tie2*, *COUP-TFII* and *APJ* mRNAs in the Flk1^{+} cells (normalized to *GAPDH* mRNA) (n=3). (**a**) The Flk1^{+} cells, transfected with control siRNA or *Tie2*-targeting siRNA were treated either with VEGF and cAMP, or with VEGF, cAMP and COMP-Ang1. *Tie2* gene expression was significantly knock-downed by the treatment with *Tie2*-targeting siRNA compared with treatment with control siRNA. (**b**) The expression level of *COUP-TFII* mRNA was upregulated by the combined treatment with VEGF, cAMP, and COMP-Ang1 when control siRNA was transfected, but not when *Tie2*-targeting siRNA was transfected. (**c**) The expression level of *APJ* mRNA was significantly upregulated by the treatment with VEGF, cAMP, and COMP-Ang1 when control siRNA was transfected, but not when *Tie2*-targeting siRNA was transfected. Values are shown as means ± SEM for three separate experiments. One-way ANOVA was used to compare differences. $^{*}P<0.05$, $^{***}P<0.001$ for the indicated groups. N.S.; not significant.

Supplementary Table 1 | *α-MHC-Cre; Ang1$^{flox/flox}$* (Ang1CKO) embryos display embryonic lethality between E12.5 and E14.5

Age at sacrifice	No. of litters analyzed	Total no.	*α-MHC-Cre; Ang1$^{flox/flox}$* (dead)	*α-MHC-Cre; Ang1$^{flox/+}$*	*Ang1$^{flox/flox}$*	*Ang1$^{flox/+}$*
E11.5	62	371	98 (2)	88	109	76
E12.5	27	191	41 (11[a])	45	53	52
E13.0	49	297	81 (23)	69	73	74
E13.5	45	282	69 (31)	65	75	73
E14.0	13	74	24 (17)	18	19	16
E14.5	12	70	14 (14[b])	17	16	16

Supplemantary Table 1 summarizes the number of embryos obtained by crossing *α-MHC-Cre; Ang1$^{flox/+}$* mice with *Ang1$^{flox/flox}$* mice.

a. On and after E12.5, dead *α-MHC-Cre; Ang1$^{flox/flox}$* (Ang1CKO) embryos were observed.

b. By E14.5, all of the Ang1CKO embryos had died.
